# Use and acceptance of traditional, complementary and integrative medicine in Germany—an online representative cross-sectional study

**DOI:** 10.3389/fmed.2024.1372924

**Published:** 2024-03-13

**Authors:** Michael Jeitler, Miriam Ortiz, Benno Brinkhaus, Mike Sigl, Rasmus Hoffmann, Miriam Trübner, Andreas Michalsen, Manfred Wischnewsky, Christian S. Kessler

**Affiliations:** ^1^Institute of Social Medicine, Epidemiology and Health Economics, Charité - Universitätsmedizin Berlin, Corporate Member of Freie Universität Berlin and Humboldt-Universität zu Berlin, Berlin, Germany; ^2^Department of Internal Medicine and Nature-Based Therapies, Immanuel Hospital Berlin, Berlin, Germany; ^3^Institute for Cultural Studies, Humboldt-Universität zu Berlin, Berlin, Germany; ^4^Institute for Sociology, Otto-Friedrich-University Bamberg, Bamberg, Germany; ^5^Faculty of Mathematics and Computer Science, University of Bremen, Bremen, Germany

**Keywords:** traditional medicine, traditional European medicine, complementary medicine, integrative medicine, alternative medicine, online-representative, cross-sectional study, Naturheilkunde

## Abstract

**Background:**

Older representative surveys show that Traditional, Complementary and Integrative Medicine (TCIM) is used by about 60% of the German population. However, no data exists for the current nationwide situation. The main aim of this cross-sectional study is to investigate the current use and acceptance of TCIM in Germany.

**Methods:**

This study is based on a representative sample of the German population aged 18–75 years. Participants were asked about the use and acceptance of TCIM. The survey was conducted online using Computer Assisted Web Interview (CAWI) in 2022 by three renowned German market research institutes on behalf of and in close coordination with the working group. The data set was analyzed descriptively and inferentially.

**Results:**

In total, 4,065 participants (52% female, 48% male, 0.4% diverse) responded completely (response rate: 21.5%). Among participants, 70% stated that they had used TCIM at some point in their lives, with 32% doing so in the last 12 months and 18% currently. The most common reason given (17%) was musculoskeletal pain. For their own health, 39% stated that TCIM is important. Traditional European Medicine was rated as very/mainly effective by 27% of participants and as partly effective by 44% (conventional medicine: 69% very/mainly effective, 19% partly effective). As a complementary treatment strategy to conventional medicine, 35% considered TCIM to be optimal (“Complementary Medicine”), 33% in combination with conventional medicine (“Integrative Medicine”) and 5% without conventional medicine (“Alternative Medicine”). The majority of the participants were in favor of more research on TCIM and stated that the costs of TCIM services should be covered by health insurance companies (71% and 69%, respectively).

**Conclusion:**

These results from a representative online-population suggest that the use of TCIM in Germany remains at a high level. The nationwide relevance of TCIM should be given greater consideration in German health care policy making. TCIM should be systematically investigated using appropriate study designs and methods including high quality randomized clinical trials to investigate their effectiveness, efficacy, therapeutic safety and costs in the future.

## 1 Introduction

According to the World Health Organization (WHO), Traditional Medicine (TM) “is the sum total of the knowledge, skill, and practices based on the theories, beliefs, and experiences indigenous to different cultures, whether explicable or not, used in the maintenance of health as well as in the prevention, diagnosis, improvement or treatment of physical and mental illness” (1). In the official WHO strategy paper “Traditional Medicine 2014–2023” and at the 1st WHO Global Summit on TM in August 2023, the WHO also calls for the increased and consistent use of these methods in primary care on a global scale (2, 3). The WHO also uses the term Traditional, Complementary and Integrative Medicine (TCIM) or Traditional, Complementary and Integrative Healthcare (TCIH) (4). We use the term TCIM as a comprehensive umbrella term here.

TM can be used as a complementary, integrative or alternative to conventional medicine (1, 2), represented in terms such as ‘Complementary Medicine’, ‘Integrative Medicine’ and ‘Alternative Medicine’. The term “Traditional European Medicine” (TEM) stands for German “Naturheilkunde” and refers to a concept of TM especially used in German speaking countries. These terms are often difficult to distinguish from one another in terms of content and the terminology used is sometimes blurred. For this reason, and to make the survey as comprehensive as possible, we decided in a consensus process to use them all while preparing the survey (5) (Table 1).

**Table 1:** Definition of the core contextual terms in the field of TCIM.

Traditional European Medicine (TEM, German: Naturheilkunde): Health promotion or treatments with natural healing methods, e.g., with phytotherapy, fasting and a healthy diet, exercise and a healthy lifestyle or Kneipp water treatments (hydrotherapy) (6).
Conventional medicine: the socially established “conventional medicine” taught at medical faculties (7).
Complementary Medicine (CM): traditional diagnostic and therapeutic methods from Western culture that complement conventional medicine, but also, for example, from traditional Chinese or Indian medicine (7).
Integrative Medicine (IM): combination of conventional medicine with evidence based Traditional European Medicine (German: Naturheilkunde) and CM (8).
Alternative medicine: non-scientifically supported healing methods which, by definition, are used as an alternative to conventional medicine, often because conventional methods are rejected (7).

The lack of clarity is also reflected in the heterogeneity of definitions and differentiations between the various methods. Historically, since the 1980s, the term ‘Complementary and Alternative Medicine’ (CAM), coined by the *National Institutes of Health* (NIH), has become widely established in the Anglo-American world. It has since been replaced by the modified term ‘Complementary and Integrative Medicine’ (CIM), which uses the word “integrative” as opposed to “alternative” to focus on the integration of evidence-informed complementary medical procedures into medical treatment as a meaningful extension (7–9). Therapeutic TCIM approaches have alongside conventional therapeutic approaches broad social acceptance in Germany (10, 11). Older surveys (Härtel 2004/Linde 2014) show that TEM was used by approximately 40–60% of the population in Germany in the 12 months prior to the respective survey (11, 12). There is a long tradition of TEM in German-speaking countries (13). In Germany, it is used in particular for health promotion and prevention, in rehabilitation, private clinics, a few inpatient facilities specializing in TEM in public hospitals and, above all, in the outpatient sector, e.g., herbal medicine, Kneipp hydrotherapy, wholefood plant-based nutrition, fasting, among others (6). Since 1988, TEM has been part of the medical licensing regulations as a cross-sectional curricular subject at medical schools (14). In March 2023, 16,118 doctors with the additional qualification of TEM were registered with the medical associations in Germany (15). Moreover, there are currently eleven appointed professors with a focus on TCIM in Germany, focusing on the scientific evaluation of TCIM according to standards of Evidence-Based Medicine (EBM) as well as the meaningful integration of such therapeutic practices into general health care (16). Reimbursement options for TCIM services by statutory health insurers are very limited in Germany. These are currently limited to acupuncture for chronic pain with the diagnosis osteoarthritis of the knee and chronic lower back pain and a very small selection of herbal remedies as well as additional selective offers from single statutory health insurance carriers like osteopathic medicine (17, 18). In the case of private health insurance, reimbursement options depend on the respective company policy. However, they are generally limited. Selected mind–body interventions (e.g., yoga) can be refunded via the so-called prevention paragraph [§20 Sozialgesetzbuch (SGB) Fifth Book (V)] in Germany.

The main aim of this population-representative survey was to update previous study results on the use and acceptance of TCIM in Germany (11, 12) and to examine additional dimensions.

## 2 Materials and methods

### 2.1 Study design

The study was conducted by the Charité University Outpatient Clinic for Complementary and Integrative Medicine at the Immanuel Hospital Berlin and the Institute of Social Medicine, Epidemiology and Health Economics of Charité—Universitätsmedizin Berlin. The study was approved by the Charité Ethics Committee and registered with ClinicalTrails.gov (NCT05530720). It was based on an online cross-sectional survey of the German resident population aged 18–80 years. The collection, processing and storage of all data generated in the study are carried out in accordance with the international guidelines for clinical trials (Declaration of Helsinki, ICH-GCP) and the research ethics framework of the accompanying sociological research.

The questions regarding the use and acceptance of TCIM were created as part of an iterative process by the working group with the involvement of TCIM experts from German-speaking countries (one professor from Switzerland, a total of six professors and four senior researchers from Germany). The final questionnaire covered the following topics: sociodemographics, use of TCIM, attitudes toward TCIM, diagnoses for which TCIM were used, importance and familiarity with terms (Supplementary material 1). This publication covers the aforementioned topics. Further items of the survey were: the role of TCIM in the context of the Covid-19 pandemic, nutrition, Ayurveda, attitude and behavior toward TCIM, Sinus milieu indicator and the EQ-5D-5L quality of life questionnaire (19, 20). Sociological analyses and a qualitative sub study are also part of the research project and will be reported elsewhere, as will other aforementioned topics of the questionnaire used in this study.

As the term TCIM is not (yet) widely used in Germany, the following four terms were used in all items of the questionnaire in German language: Traditional European Medicine (German: Naturheilkunde), Complementary Medicine, Integrative Medicine as well as Alternative Medicine. However, the umbrella term TCIM is used throughout the results section.

The survey was implemented by three renowned German market research institutes (Conversio, Sinus Institute and Respondi Institute) on behalf of and in close coordination with the study management. Conversio advised on the methodology and feasibility of the questionnaire for an online survey and commissioned the other two institutes and supervised the survey process. Sinus contributed the institute’s own national milieu indicator, while Respondi carried out the online survey. The respondents were recruited from Respondi’s online access panel but remained completely anonymous.

The inclusion criteria for the study included active consent to the informed consent form, a minimum age of 18 years, sufficient German language skills and sufficient cognitive abilities to take part in an online survey lasting approximately 30 min. Exclusion criterion was a lack of consent to participate in the study.

The survey was conducted online using Computer Assisted Web Interview (CAWI). The online panel is certified according to the international standard ISO 26362, which monitors the quality of online sampling. This includes quality procedures that continuously check the response behavior. In general, studies based on probability samples, in which each member of the population has a known and non-zero chance of being included in the sample, are preferred to non-probability samples (21). In order to come as close as possible to the requirements of a representative sample, participation in Respondi requires a double opt-in registration, with a team of experts monitoring and managing the panel. The number of Respondi panelists contains approximately 100,000 participants with good coverage of different age, education and income groups. A key advantage of an online access panel is the experience and motivation of the panel participants, which means that high data quality can be achieved even for complex questions. The online mode also offers the advantage that questions are answered more truthfully, particularly with regard to the sensitive area of health.

The quotas were based on the best4planning (B4P) study, which follows the methodological standard for drawing representative samples (22). With B4P, a structural analysis of the German resident population between the ages of 18 and 80 was carried out to determine the quota specifications for the study population. The B4P study itself is based on a sample of more than 30,000 randomly selected people. Quota control makes it possible to compensate for socio-demographic imbalances in relation to the overall population.

### 2.2 Statistical analysis

As part of the descriptive analyses, (relative) response frequencies and various measures of position (e.g., mean, median) and dispersion (e.g., standard deviation, variance) were described in the results for the overall sample as well as for various subgroups. Crosstab analysis helped to make informed decisions by identifying patterns, correlations, and trends between the study’s parameters. Decision trees were calculated using Exhausted Chi-squared Automatic Interaction Detection (CHAID) or Classification and Regression Trees (CRT). The data were analyzed using R (R Foundation; version 4.3) and IBM SPSS Statistics (vers. 29). Data were unweighted or, if weighted, then based on age, gender, education, federal state and city size. As the weighted and unweighted values differ only in the decimal places, except for the sociodemographic characteristics, we only report the unweighted values in the results. Both the unweighted and weighted values are shown in the sociodemographic characteristics (Table 2).

**Table 2:** Sociodemographic characteristics of the study population (*n* = 4,065).

	Unweighted		Weighted	
	*n*	%	*n*	%
Gender				
Male	1947	47.9	2026	49.8
Female	2,101	51.7	2018	49.6
Diverse	17	0.4	21	0.5
Age in years				
Under 20	67	1.6	87	2.1
20 to 29	557	13.7	679	16.7
30 to 39	631	15.5	701	17.2
40 to 49	656	16.1	651	16.0
50 to 59	896	22	863	21.2
60 to 75	1,258	30.9	1,084	26.7
Education				
No general school-leaving certificate (yet)	30	0.7	36	0.9
Secondary (elementary, basic) school leaving certificate without completed apprenticeship/vocational training	251	6.2	271	6.7
Secondary school leaving certificate with completed apprenticeship/vocational training	885	21.8	891	21.9
Secondary school without A-levels (German: Realschulabschluss/Mittlere Reife/Oberschule) or equivalent qualification	1,173	28.9	1,288	31.7
A-levels, (technical) university entrance qualification without studies	751	18.5	723	17.8
Studies (university, college, university of applied sciences, polytechnic)	949	23.3	835	20.6
PhD	26	0.6	19	0.5
Federal states				
Baden-Wuerttemberg	417	10.3	537	13.2
Bavaria	604	14.9	630	15.5
Berlin	342	8.4	174	4.3
Brandenburg	98	2.4	122	3
Bremen	40	1	33	0.8
Hamburg	159	3.9	89	2.2
Hesse	293	7.2	305	7.5
Mecklenburg-Western Pomerania	71	1.7	78	1.9
Lower Saxony	341	8.4	403	9.9
North Rhine-Westphalia	868	21.4	878	21.6
Rhineland-Palatinate	179	4.4	203	5
Saarland	43	1.1	49	1.2
Saxony	238	5.9	199	4.9
Saxony-Anhalt	107	2.6	106	2.6
Schleswig-Holstein	152	3.7	146	3.6
Thuringia	113	2.8	114	2.8
Personal monthly net income				
No own income	175	4.3	199	4.9
Up to 1,000 €	887	21.8	883	21.7
1,000–2000 €	1,549	38.1	1,544	38
2000–3,000 €	959	23.6	952	23.4
3,000–4,000 €	311	7.7	317	7.8
4,000–5,000 €	99	2.4	93	2.3
> 5,000 €	85	2.1	76	1.9
Net monthly household income				
Up to 1,000 €	505	12.4	482	11.9
1,000–2000 €	1,047	25.8	1,023	25.2
2000–3,000 €	1,049	25.8	1,051	25.8
3,000–4,000 €	735	18.1	769	18.9
4,000–5,000 €	424	10.4	443	10.9
> 5,000 €	305	7.5	298	7.3
Location size				
Under 2,000 inhabitants	273	6.7	310	7.6
2,000 to under 5,000 inhabitants	232	5.7	267	6.6
5,000 to under 20,000 inhabitants	603	14.8	1,072	26.4
20,000 to under 50,000 inhabitants	557	13.7	655	16.1
50,000 to under 100,000 inhabitants	401	9.9	470	11.6
100,000 to under 500,000 inhabitants	889	21.9	617	15.2
500,000 inhabitants and more	1,110	27.3	674	16.6
Religious community				
Catholic	853	21.0	894	22.0
Protestant	1,051	25.9	1,096	27.0
Muslim	69	1.7	70	1.7
Buddhist	17	0.4	18	0.4
Hindu	2	0.1	2	0.1
Jewish	12	0.3	8	0.2
Other	72	1.8	68	1.7
No religious affiliation/atheist	1989	48.9	1909	47.0
Party affiliation				
CDU	506	12.4	515	12.7
CSU	177	4.4	184	4.5
FDP	229	5.6	244	6
Bündnis 90/Die Grünen	637	15.7	595	14.6
SPD	650	16	629	15.5
Die Linke	330	8.1	311	7.6
AfD	418	10.3	443	10.9
Other political parties	194	4.8	206	5.1
Not specified	924	22.7	941	23.1
Medical background				
Nursing training/nursing studies	195	4.8	193	4.7
Alternative practitioner examination	39	1	42	1
Medical studies	51	1.3	57	1.4
Pharmacy studies	29	0.7	32	0.8
Physiotherapy training	39	1	43	1.1
Occupational therapy training	27	0.7	27	0.7
Self-acquisition of basic medical knowledge	670	16.5	670	16.5
Miscellaneous	112	2.8	110	2.7

PhD, Philosophiae Doctor; CDU, Christlich Demokratische Union Deutschlands; CSU, Christlich-Soziale Union; FDP, Freie Demokratische Partei; SPD, Sozialdemokratische Partei Deutschlands; AfD, Alternative für Deutschland.

## 3 Results

The survey was conducted from September to October 2022; 41,011 invitations were sent out. Of these, 8,821 participants started the survey (response rate 21.5%). Based on the exclusion criteria (mainly due to having read study information but not giving consent, no age information), 453 cases were removed. Additionally, 2,845 participants were excluded because they had already assigned themselves to closed quotas. Exactly 1,000 people dropped out of the survey and were therefore not included in the analysis. A total of 4,505 participants completed the questionnaire. In the quality screening, 18 participants were excluded due to variance check in the Sinus-Milieu indicator. Subsequently, a further 295 participants were removed for quality reasons, including conspicuous open-ended responses and using the quality variable. The final dataset included 4,210 participants. In order not to compromise the online representativeness due to the upper age limit of 80 years, this was reduced to 75 years. As a result, the final population-representative data set for the age group 18–75 years comprises a total of 4,065 participants (nearly 10% of the invited persons; 51.7% female, 47.9% male, 0.4% diverse; average age: 49.3 ± 15.8 years). Further sociodemographic characteristics are listed in Table 2.

### 3.1 Use of TCIM

**Figure 1:**
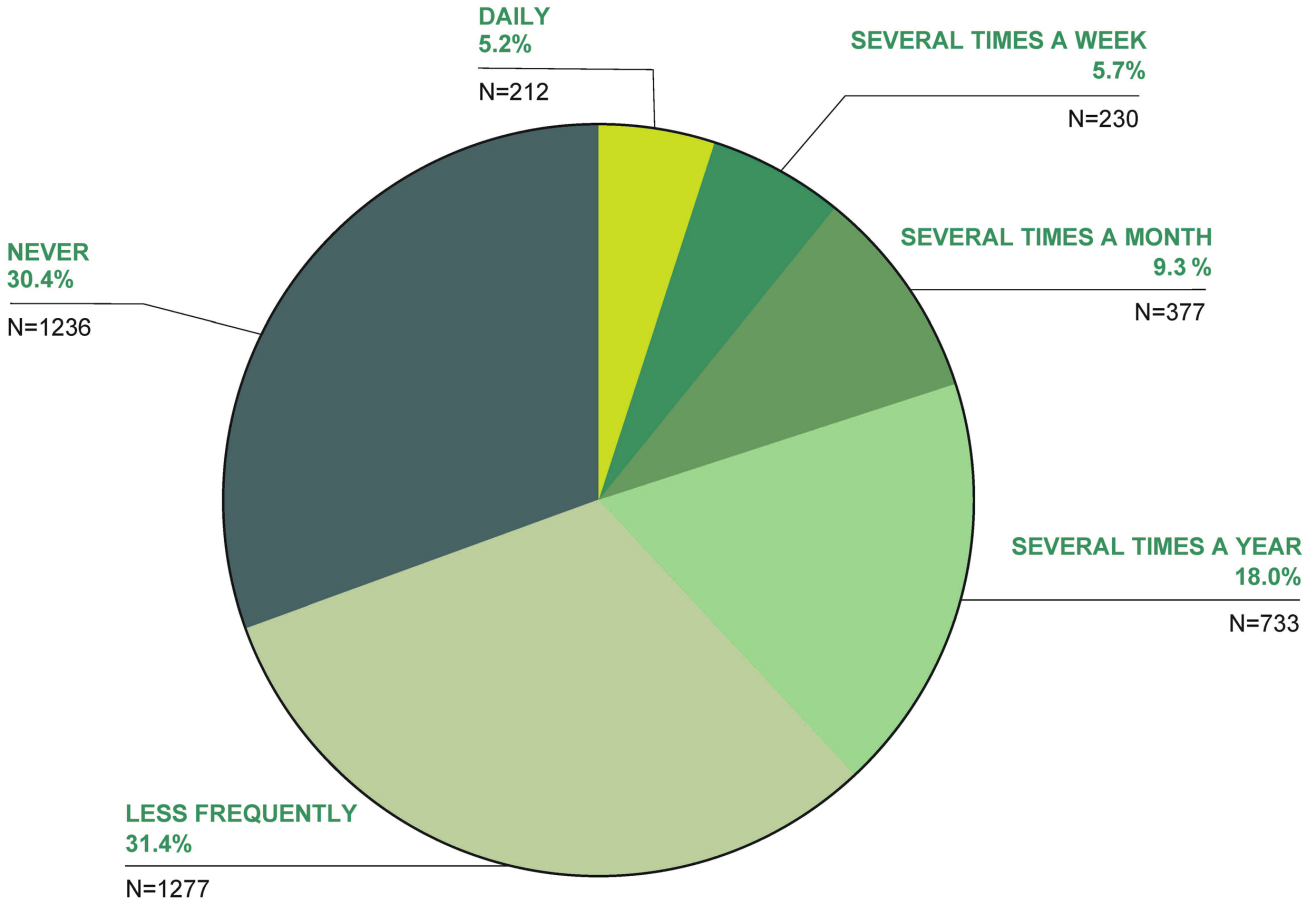
How often do you currently use TCIM?

The use of TCIM is shown in Figure 1; 69.6% of participants stated that they had used TCIM at some point. At the time of the survey, 10.9% of participants used TCIM daily or several times a week (9.3% several times a month, 18% several times a year, 31.4% less frequently and 30.4% never; Figure 1).

In addition, 31.8% of participants had used TCIM in the last 12 months (63.3% had not, 4.9% did not know). At the time of the survey, 17.5% were “currently” (at the time of the survey) using TCIM, with 78.5% not doing so and 4% did not know. Regarding future intentions, 38.1% intended to use TCIM, while 40% did not know and 21.9% had no intention. Additionally, 14.8% stated that other household members were currently using TCIM, with 78.4% unsure and 6.8% who did not know.

Regarding the sociodemographic characteristics of TCIM use in the last 12 months, the parameters gender, age, education, net monthly household income, religious community and party affiliation differed highly significantly in both the unweighted and weighted dataset (each *p* = 0.001; Supplementary Table 1 in Supplementary Material 2). Only the personal monthly net income differed significantly regarding the use of TCIM in the last 12 months in the weighted data set (*p* = 0.037).

TCIM was used more frequently by women in the last 12 months (women 37.3%, men/diverse 25.9%, from the whole sample, *p* < 0.001; Supplementary Figure 1 in Supplementary Material 2). The proportion of TCIM use in the last 12 months was significantly dependent on the level of education. Participants with at least a high school diploma had used TCIM in the last 12 months at a rate of 36.7%; without a high school diploma, TCIM use was at 28.1% (*p* < 0.001). Almost every second woman (46.5%) with at least a high school diploma and a total monthly income of over €2,000 had used TCIM in the last 12 months.

If these women classified themselves as spiritual, the percentage increased to 64.6%. Familiarity with TCIM correlated highly significantly with its use in the last 12 months for women and men (*p* < 0.001), but there are still gender differences: 74.1% of women and 64.0% of men who were very familiar with TCIM had also used TCIM in the last 12 months.

Household income is a significant (*p* < 0.001) predictor for the use of TCIM in the last 12 months. A maximum of 38.2% was found in the group with a monthly household income of 4,000-5,000€. In the group with a household income of up to 500€, the proportion was still 19.6%. Own income, on the other hand, was only significant for men (*p* = 0.001; Supplementary Figure 1 in Supplementary Material 2). In the last 12 months, 36.6% of men with an income of over 4,000€ had used TCIM. For those who earned up to 2,000€ it was 22.8% (Supplementary Figure 1 in Supplementary Material 2).

### 3.2 Diagnoses for which TCIM was used

**Figure 2:**
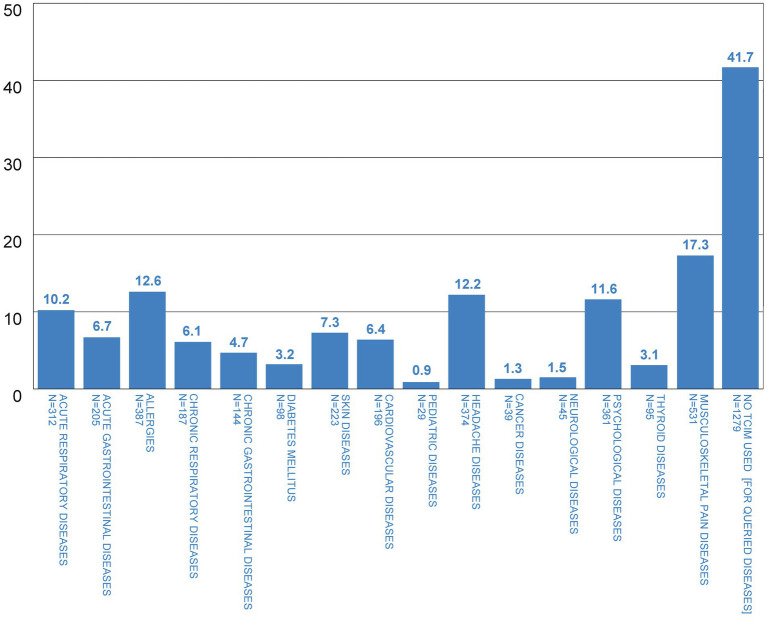
For which illnesses have you already used TCIM? (in %).

**Figure 3:**
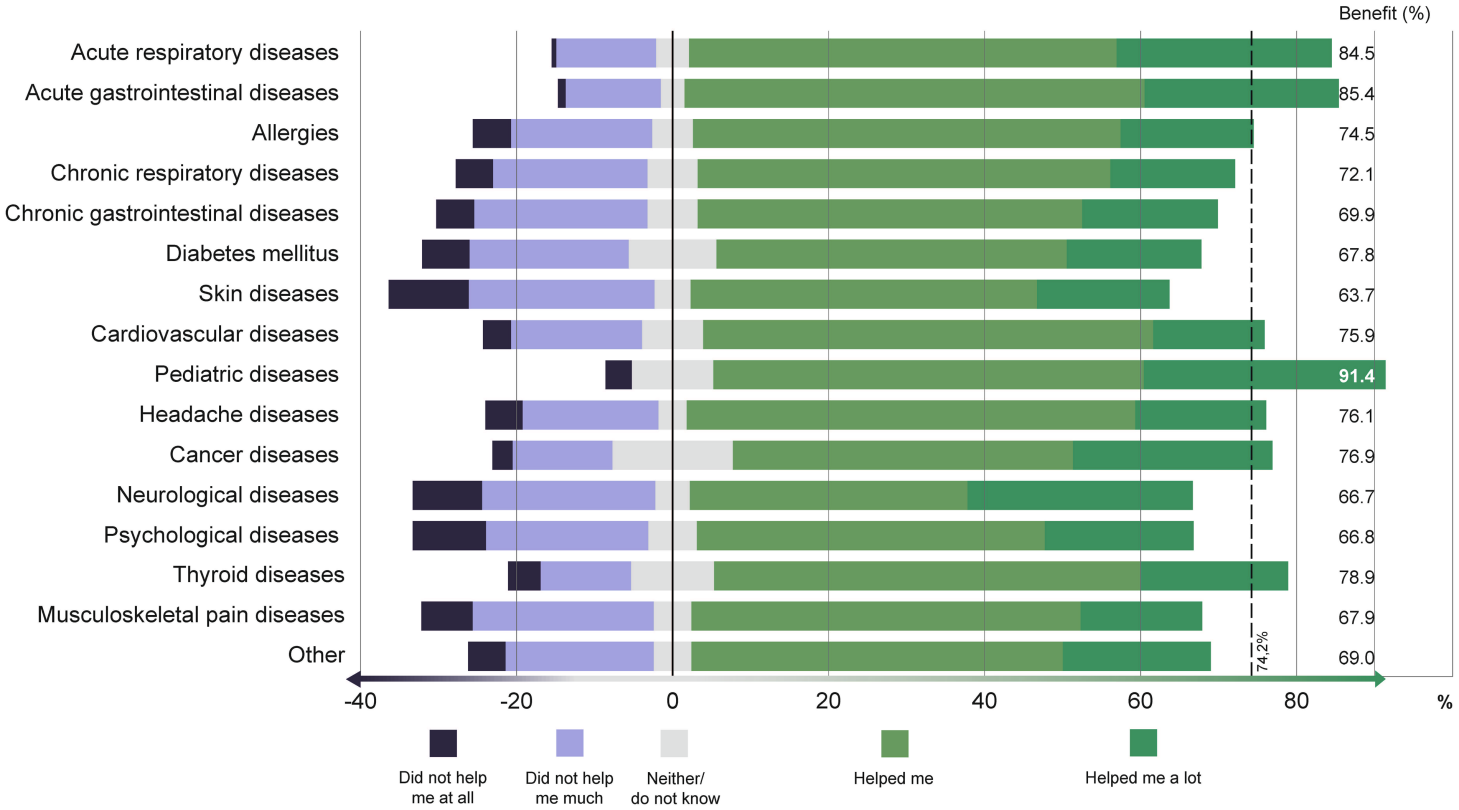
To what extent has TCIM helped you with the following illnesses? (in %).

TCIM was used most frequently for musculoskeletal pain diseases (17.3%), followed by allergies (12.6%), headache (12.2%), psychological diseases (11.6%) and acute respiratory diseases (10.2%); 41.7% stated that they had not used TCIM at all (for queried diseases) (Figure 2). TCIM predominantly helped or helped the participants a lot with pediatric diseases (91.4% benefit, i.e., ‘helped me’ or ‘helped me a lot’), acute gastrointestinal diseases (85.4% benefit) and acute respiratory diseases (84.5% benefit; Figure 3). TCIM was used with minimal benefit in skin diseases (63.7% benefit), neurological and psychological diseases (66.7 and 66.8% benefit respectively).

**Figure 4:**
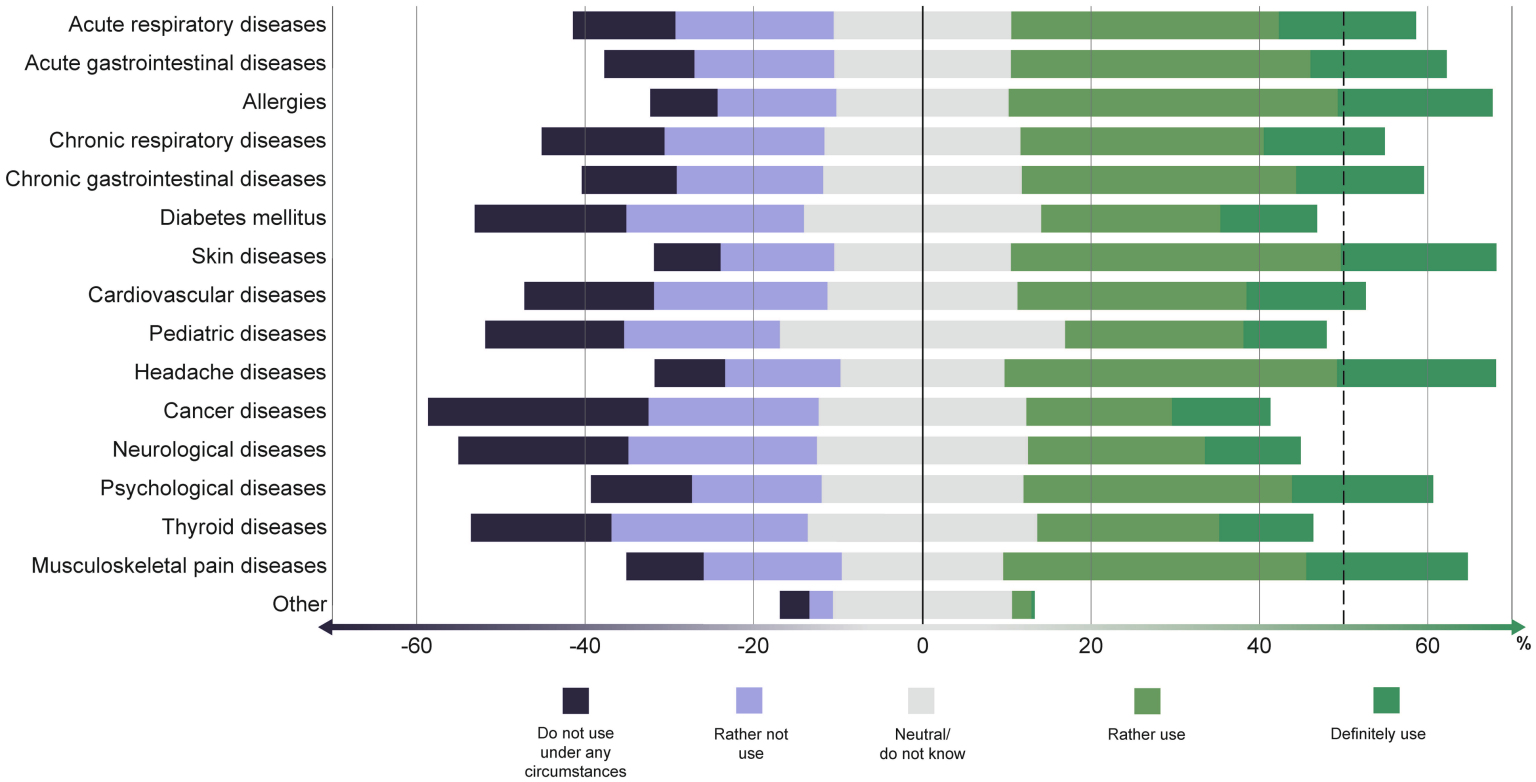
To what extent would you use TCIM for the following illnesses? (in %).

When being asked for hypothetical uses, the participants referred that they would use TCIM primarily for headaches, skin diseases, allergies, musculoskeletal pain diseases, acute gastrointestinal diseases, and psychological diseases (Figure 4). For cancer, pediatric diseases, neurological diseases, diabetes mellitus and thyroid diseases, TCIM would be used by fewer participants on average, although the absolute differences are small.

Dietary supplements, vitamin supplements, or herbal remedies were taken daily by 41.9% of respondents. Just over half of the respondents (50.4%) took conventional medication daily.

**Figure 5:**
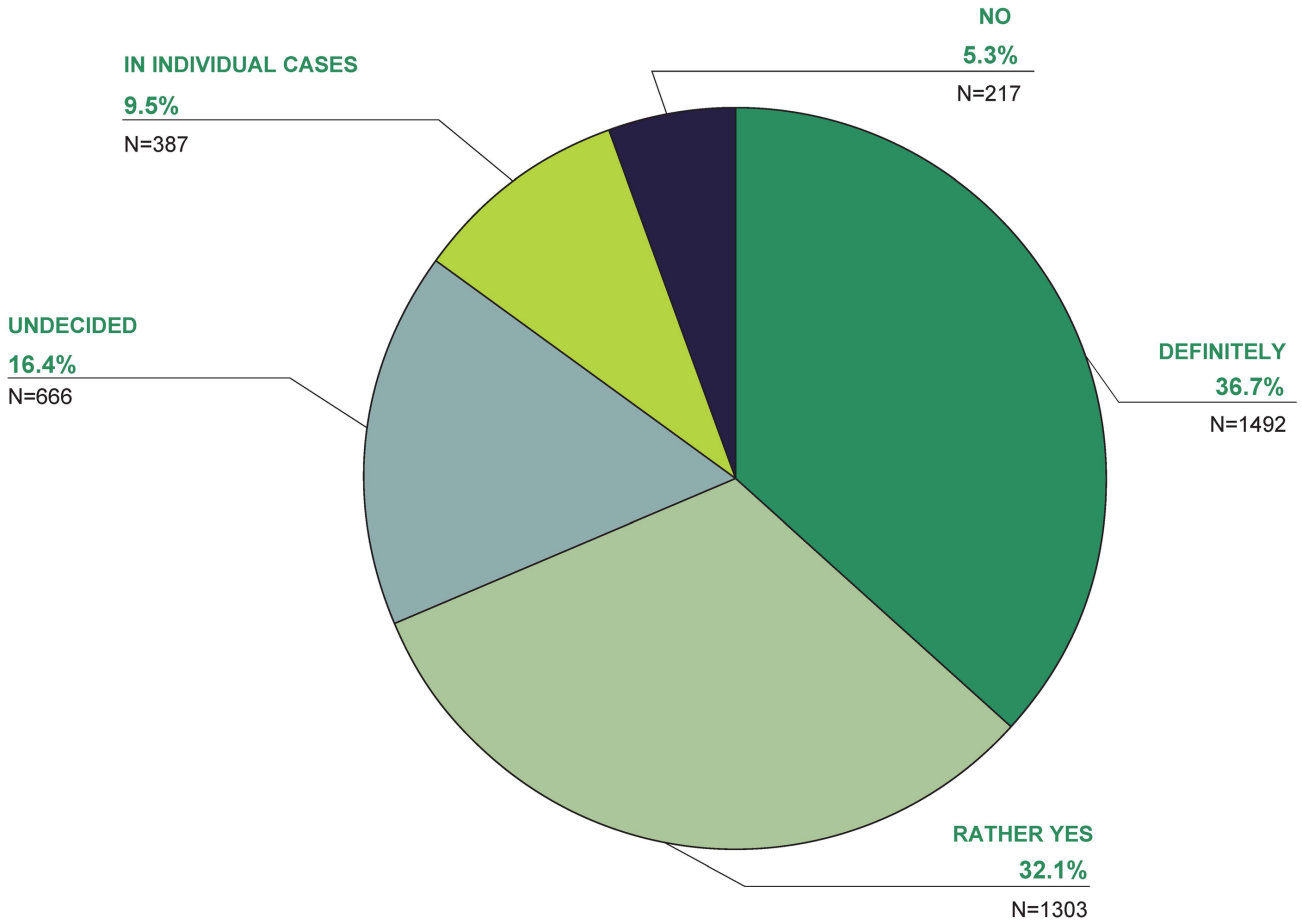
In your opinion, which of the following answers is correct? The costs of TCIM services should be covered by health insurance.

The majority are in favor (36.7%: definitely, 32.1%: rather yes) of the costs of TCIM services being covered by health insurance (16.4% were undecided, 9.5% stated in individual cases, 5.3% rejected this; Figure 5).

### 3.3 Importance and familiarity with terminology

**Figure 6:**
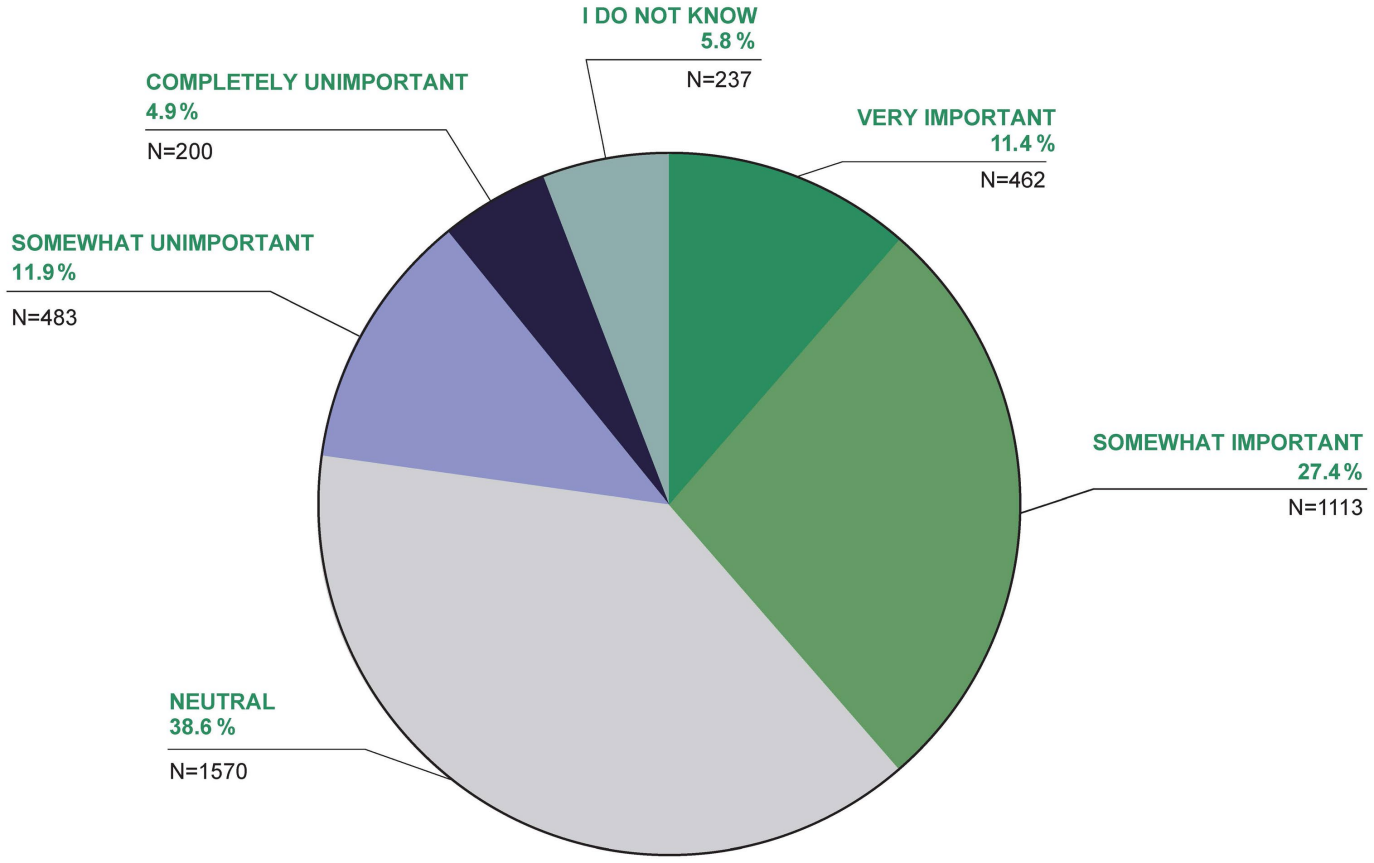
How important is TCIM for your health?

For 38.8% of the participants, TCIM was very important or somewhat important for their own health, 16.8% felt that TCIM was somewhat or completely unimportant for their own health (38.6% were neutral, 5.8% did not know; Figure 6).

**Figure 7:**
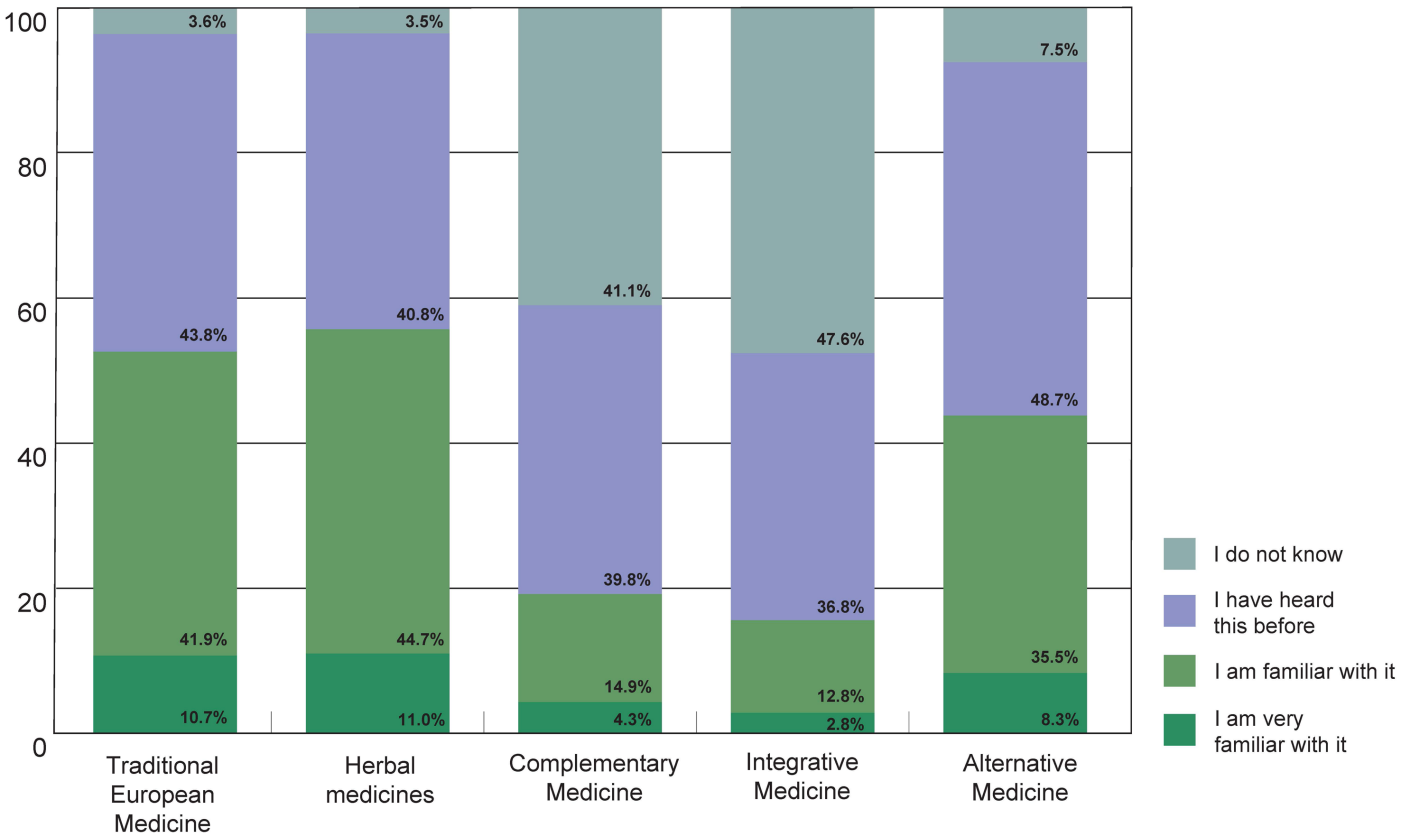
To what extent are you familiar with the following terms?

When asked to what extent the participants were familiar with terms related to TCIM, the terms Traditional European Medicine (German: Naturheilkunde), herbal medicines and Alternative Medicine were particularly familiar (Figure 7). The terms Complementary Medicine and Integrative Medicine, which are mainly used in an academic context in Germany, were less familiar.

In terms of familiarity with TCIM procedures, acupuncture (96.7%), fasting (94.6%), homeopathy (95.1%) and yoga (95.8%) were the best-known procedures (Supplementary Figure 2 in Supplementary Material 2). The better-known methods include various other methods, e.g., Ayurveda, hydrotherapy/water treatments/Kneipp baths, phytotherapy/herbal medicine, manual medicine/osteopathy/chiropractic care, traditional Chinese medicine, whole-food plant-based nutrition, stress management/relaxation methods/meditation (Mind–Body Medicine), movement/dance therapy, art/music therapy. Less well-known methods were anthroposophic medicine, Hijama, traditional African medicine and forest bathing/forest therapy (Supplementary Figure 2 in Supplementary Material 2).

### 3.4 Attitudes toward TCIM

**Figure 8:**
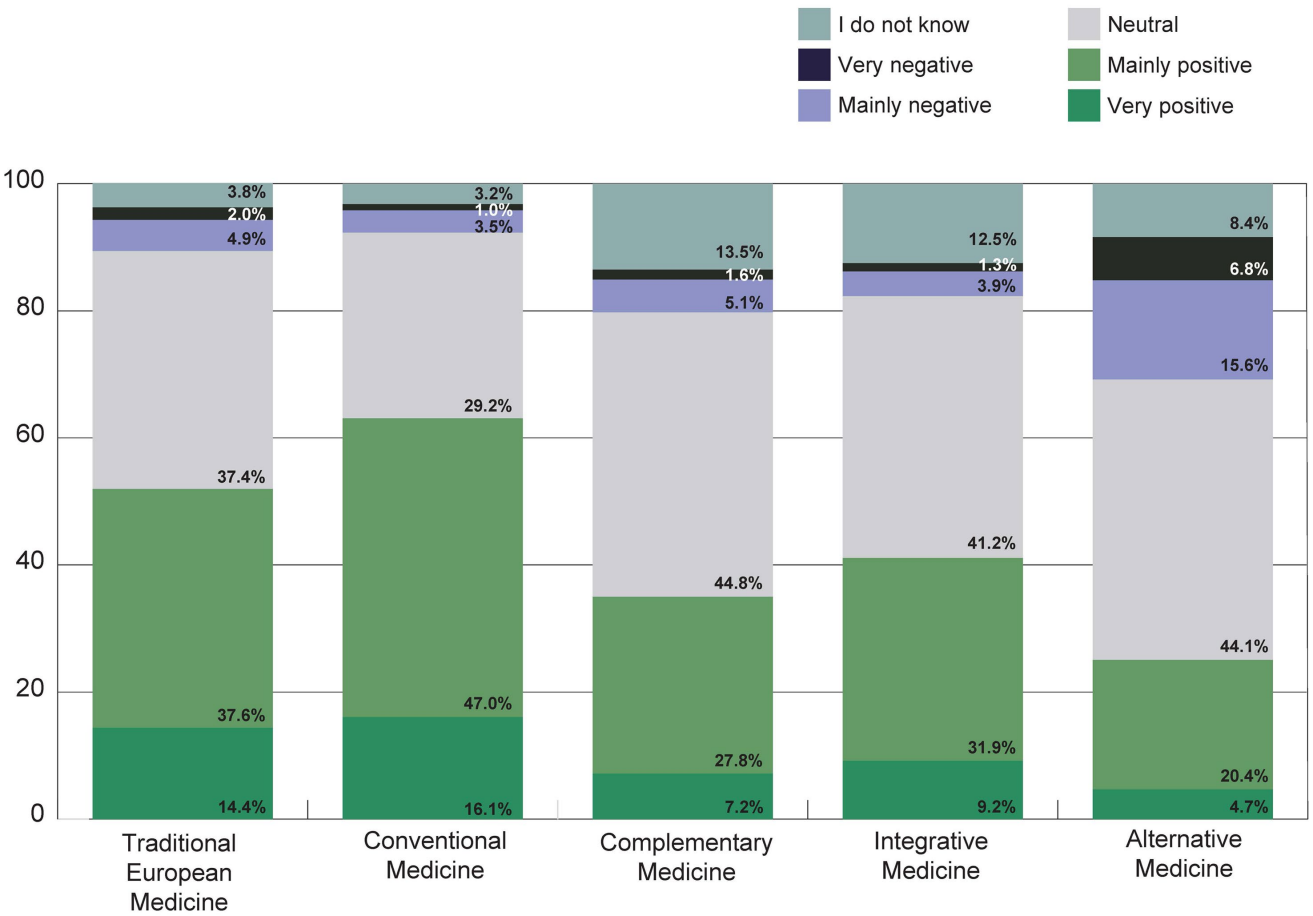
How is your general attitude toward Traditional European Medicine (German: Naturheilkunde), conventional medicine, Complementary Medicine, Integrative Medicine or Alternative Medicine? The terms were explained directly with this question (see Table 1).

The attitude toward Traditional European Medicine (German: Naturheilkunde) was very or predominantly positive in 52% of the participants, and 63.1% toward conventional medicine (Figure 8). Integrative Medicine and Complementary Medicine were perceived as very or predominantly positive by 41.1 and 35%, respectively. Alternative Medicine was rated as very or mostly positive by only a quarter (25.1%) of respondents, while 22.4% rated Alternative Medicine as mostly or very negative (Figure 8).

**Figure 9:**
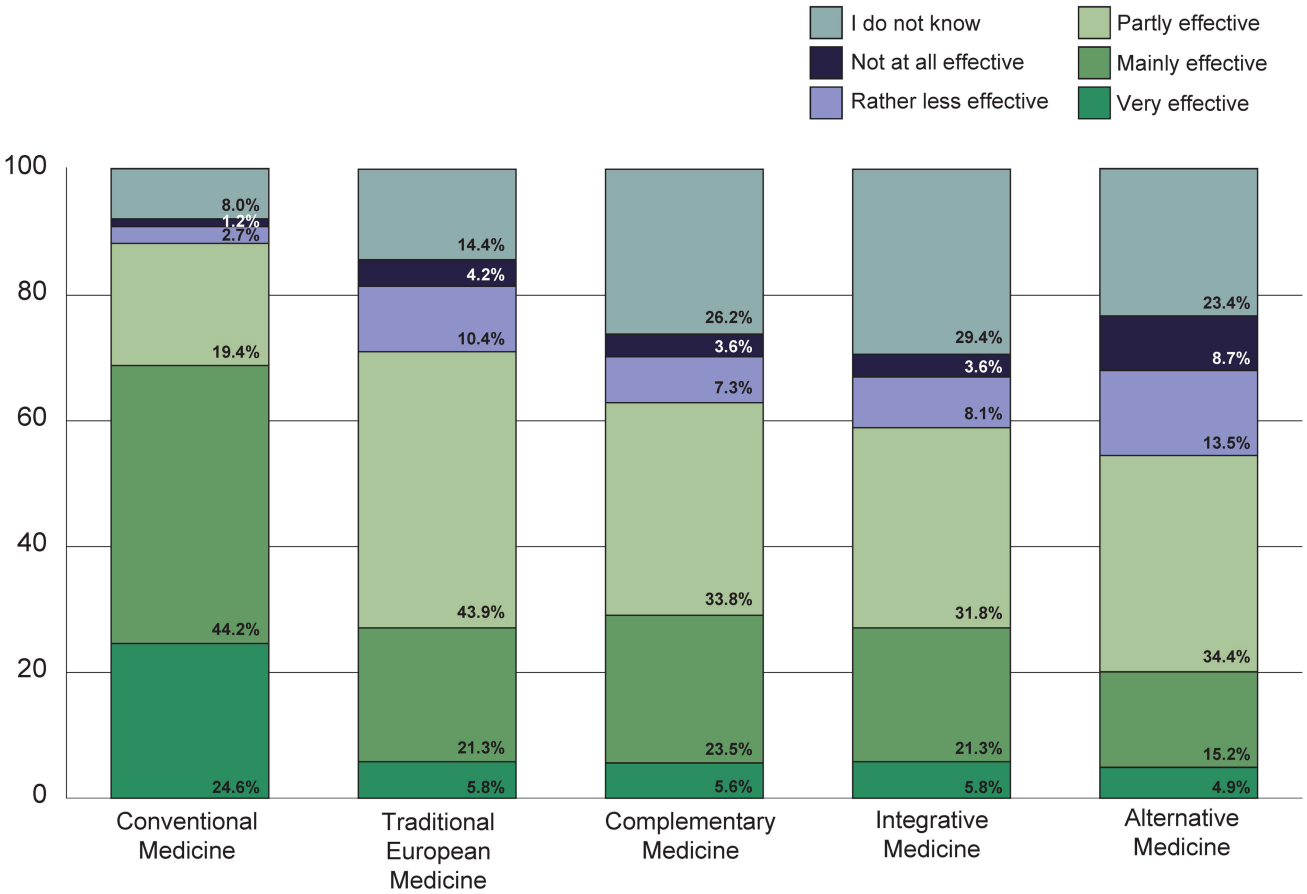
How effective do you think the following medical procedures are?

Conventional medicine was rated as very or mostly effective by over two thirds of the participants, while Traditional European Medicine, Complementary Medicine and Integrative Medicine were rated as very or mostly effective by around a quarter, and Alternative Medicine by around a fifth of the respondents (Figure 9).

**Figure 10:**
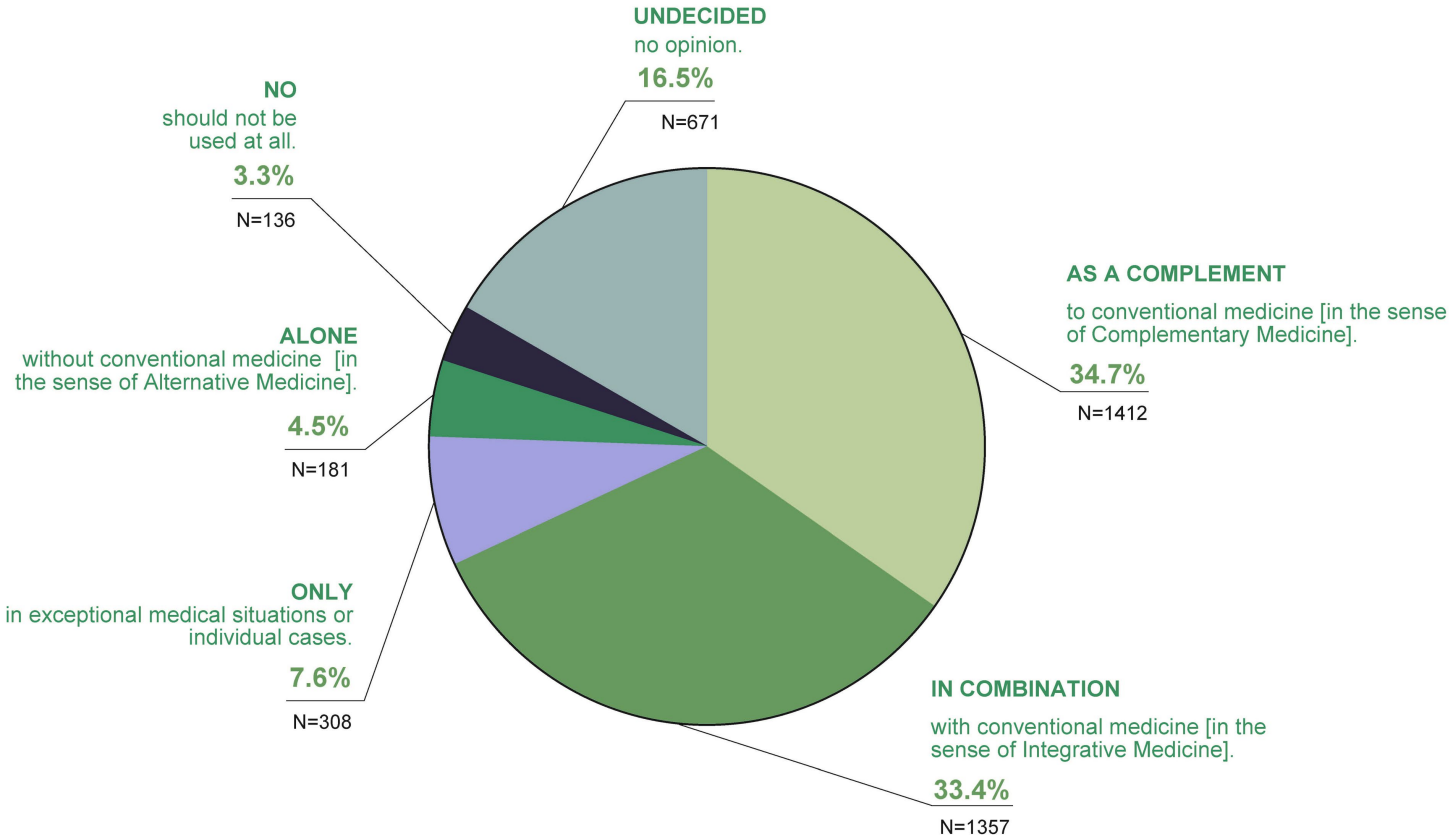
To what extent do you consider TCIM to be optimal?

When asked to what extent the participants considered TCIM to be optimal (in terms of integration into the healthcare system), 34.7% stated that they used it as a supplement to conventional medicine (in the sense of Complementary Medicine), 33.4% in combination with conventional medicine (in the sense of Integrative Medicine; Figure 10).

Stated opinions varied: 7.6% stated TCIM to be only optimal in exceptional medical situations or individual cases, 4.5% alone, without conventional medicine (in the sense of Alternative Medicine).

**Figure 11:**
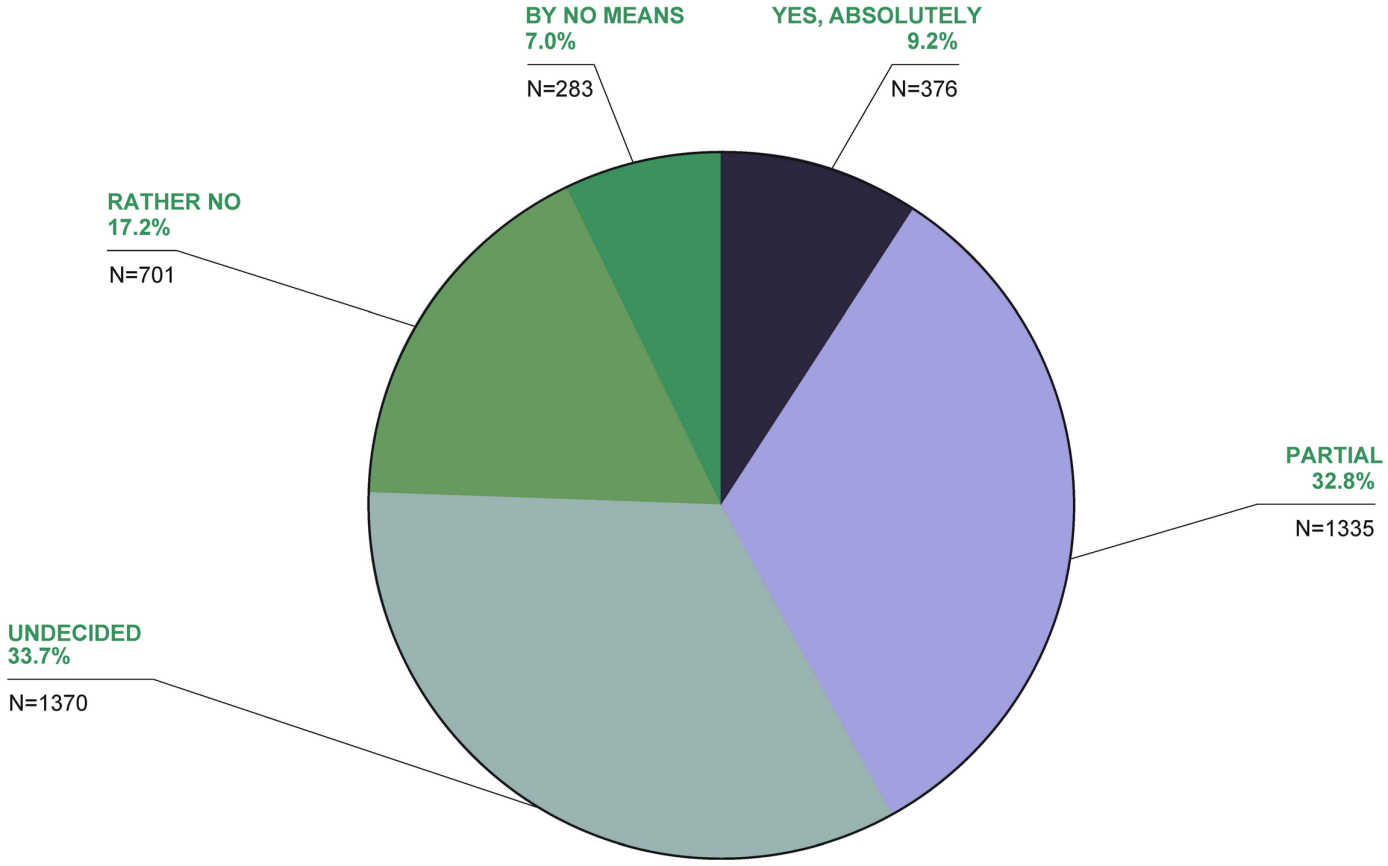
TCIM is often described as unscientific. Would you agree with this assessment in principle?

**Figure 12:**
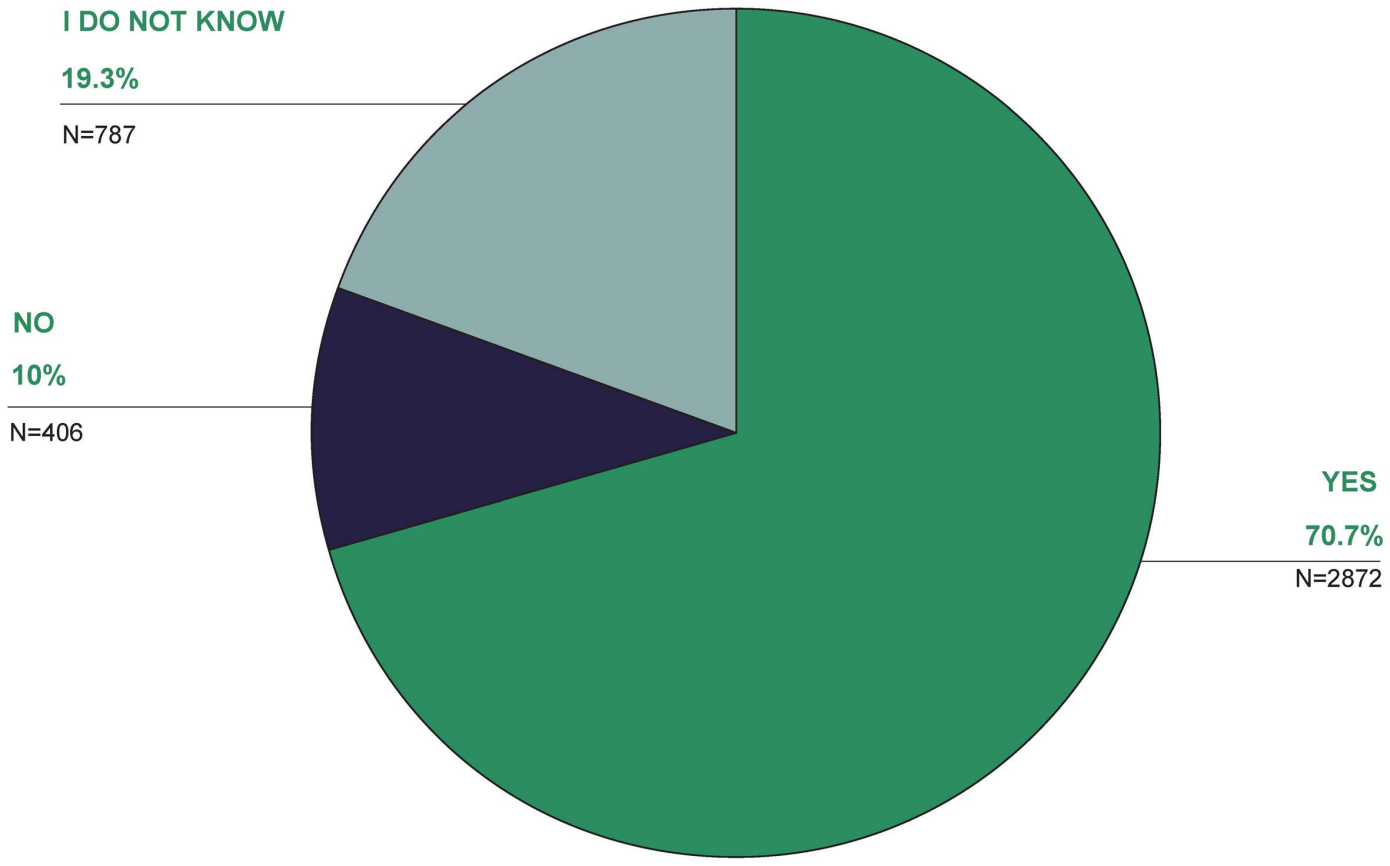
In your opinion, should there be more research into TCIM?

**Figure 13:**
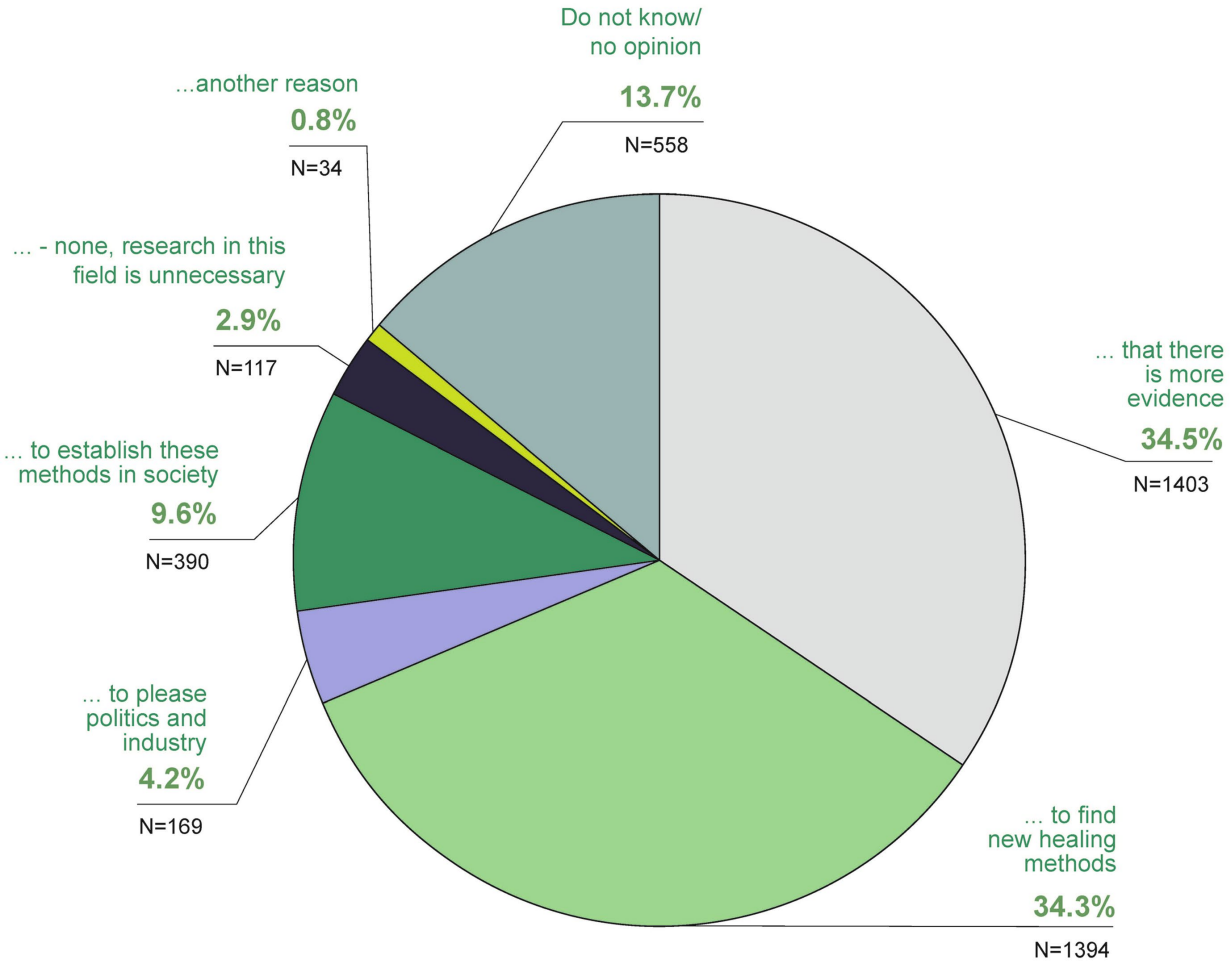
In your opinion, which of the following answers is correct? In my opinion, the main reason for research in the field of TCIM is above all.

Another 3.3% believed TCIM should not be used at all, with 16.5% undecided or having no opinion. Regarding its scientific credibility, 9.2% agreed TCIM is unscientific, with 32.8% partially agreeing, 33.7% undecided, 17.2% rather no and 7% not at all (Figure 11). Around a third of the respondents (32.8% partly, 33.7% undecided) agreed with this statement to some extent or were undecided. 17.2% of the participants voted rather no and 7% strongly disagreed with the statement. Over two thirds (70.7%) were in favor of more TCIM research (Figure 12). The main reasons for research in the field of TCIM for 34.5% were that there should be more evidence and for 34.3% to find new healing methods (Figure 13).

**Figure 14:**
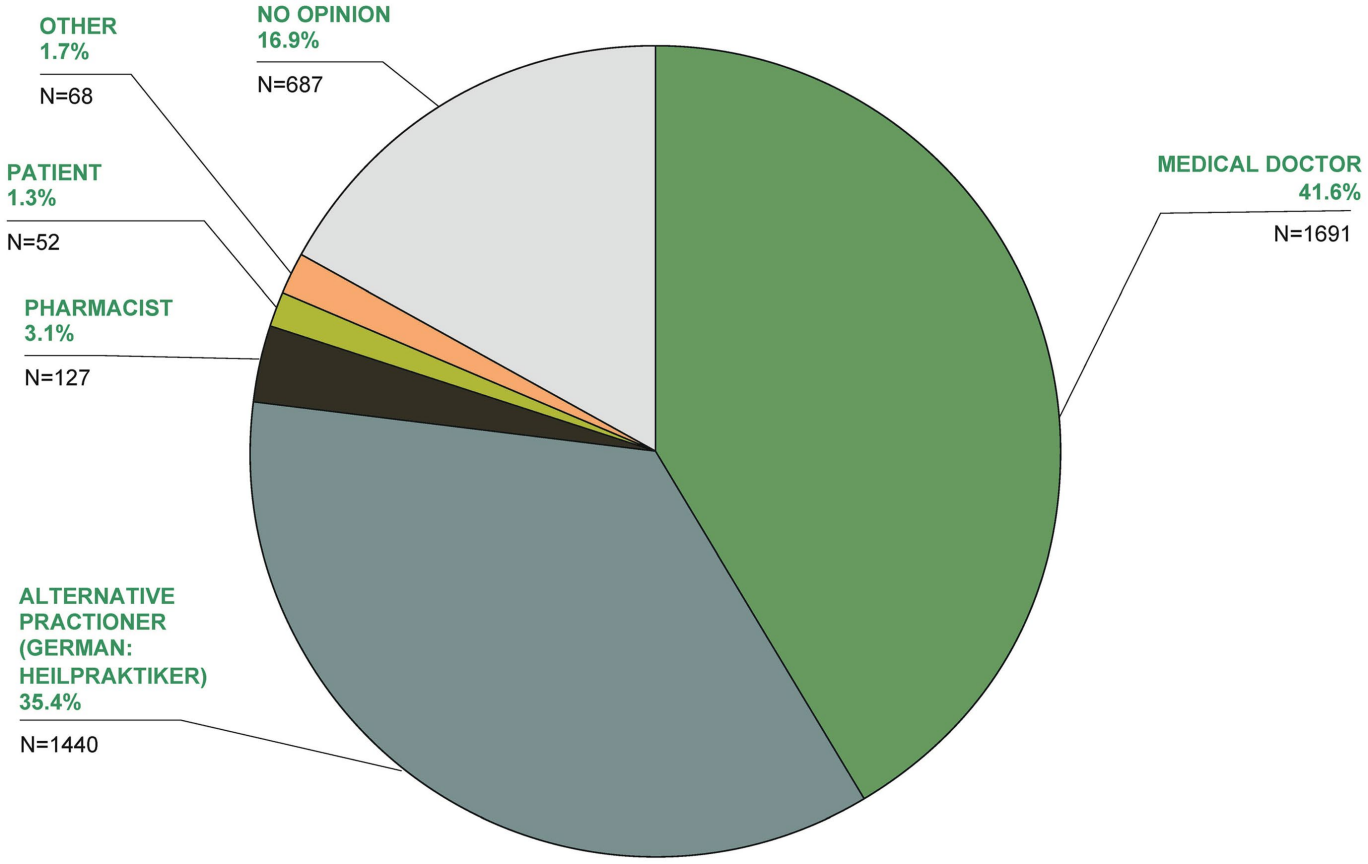
In your opinion, who has the greatest expertise in the field of TCIM?

The greatest expertise in TCIM, according to 41.6% of respondents, was stated to be held by medical doctors, followed by alternative practitioners (German: Heilpraktiker; 35.4%; Figure 14).

**Figure 15:**
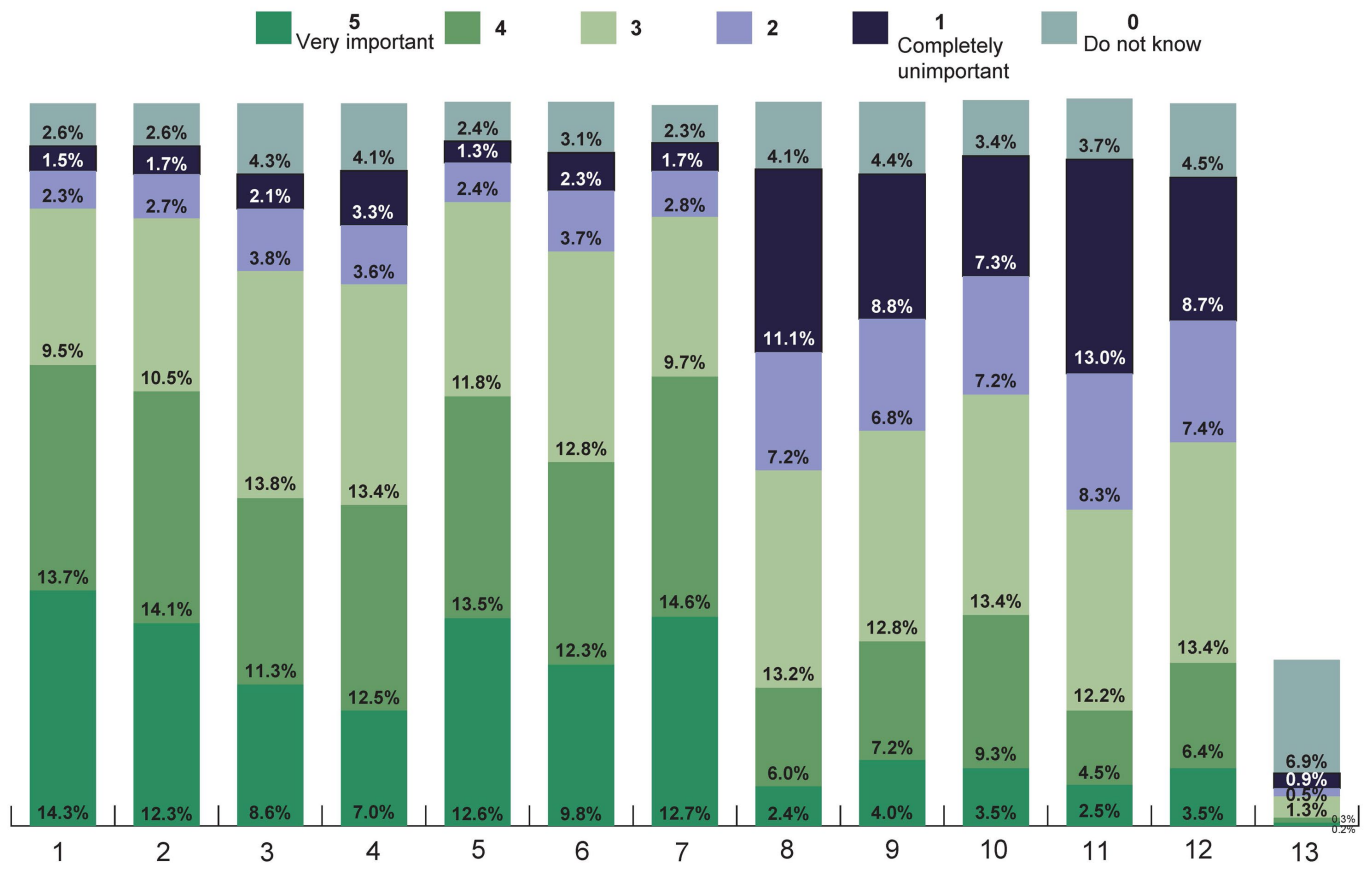
How important are the reasons listed for using TCIM to you (Likert scale 1–5 from 1 = completely unimportant to 5 = very important)? Listed reasons were the following: 1 Fewer side effects than with conventional medicine, 2 To reduce the side effects of conventional medicine, 3 Chances of recovery are better, 4 Family, friends or acquaintances have had good experiences, 5 I myself have had good experiences, 6 This strengthens my health competence/self-treatment competence, 7 The advice of my treating doctor, 8 I have heard about it in the media, 9 I am doing it out of health-related desperation, 10 I am doing it out of curiosity about these procedures, 11 I do not think much of conventional medicine, 12 I have had bad experiences with conventional medicine, 13 Other.

From the selection of 12 reasons given for using TCIM, the main reasons given were to have fewer side effects than with conventional medicine (very important: 14.3%), reduction of side effects of conventional medicine (very important: 12.3%), former own positive experiences (very important: 12.6%) and the advice of the treating doctor (very important: 12.7%; Figure 15).

In particular, medical recommendations and previous personal experience influenced the decision to choose a treatment method, followed by the results of scientific studies, experiences of family, friends and acquaintances as well as personal recommendations (Supplementary Figure 3 in Supplementary Material 2).

## 4 Discussion

More than 10 years after the last representative survey on this topic, this study provides a population-representative online-survey update on the use and acceptance of TCIM in Germany. Out of the 4,065 participants, almost 70% stated that they had used TCIM at some point in their lives, with 32% doing so in the last 12 months and 18% currently. The results suggest that TCIM is known about, valued and socially anchored nationwide across all socio-structural characteristics surveyed. It is interesting to note a “base phenomenon,” i.e.,—depending on the question—approx. 25–35% make use of TCIM in Germany. Only a small proportion of participants considered these medical procedures to be ineffective, while most respondents would like the costs of TCIM services to be paid by health insurance providers. A large proportion of respondents were in favor of more intensive research in the field of TCIM. Regarding individual procedures, there are considerable differences in the population, e.g., yoga and fasting as more familiar procedures and forest therapy and anthroposophic medicine as more unknown procedures.

### 4.1 Strengths and limitations

Limitations include a low response rate of 21.5%, which may call into question the generalizability of the results. To increase generalizability, the data used for the analysis was weighted according to age, gender, education, federal state and city size. However, the weighted and unweighted data hardly differed from each other, which suggests a sufficient quota system. The study is based on a survey that was conducted using an online access panel. The basis for this decision on data collection was the high-quality standard that is guaranteed in the selection and maintenance of the participants of the access panel used, as well as the use of a quota system (21). These aspects facilitate the articulation of population-represetative statements about the use and acceptance of TCIM within the German population. The online mode proofed to be particularly suitable for this research, for it offered a space of discretion and sesitivity regarding questions on personal health. It should be noted, however, that the use of an access panel meant that special populations, such as those without online access and with a low online affinity, were excluded. However, the exclusion of special populations is not a specific problem of access panels or online surveys in general. Telephone surveys and written surveys also exclude people without a telephone connection or home address. An additional difficulty with telephone and written surveys is that they are tied to the time and place of the survey, which is largely not the case with online surveys. The limitation of the very elderly possibly not being represented in this study, as they are less likely to have internet access, had little impact, as this age group was excluded from our analysis sample.

### 4.2 Comparison with other trials

Due to very heterogeneous survey methods in previous surveys on the topic and a great variety in terms of definitions and terminology of unconventional healing methods as well as traditional medical systems such as Chinese medicine and Ayurveda, it is problematic to compare the results directly with each other (11, 12). In one of the few representative survey studies of 1,100 participants published in peer-reviewed journals in 2004 by Härtel/Volger, approximately 62% had used at least one TCIM intervention in the 12 months prior to the survey (11). In a population-representative online panel survey in Switzerland of 6,375 people aged 16 and above, almost two-thirds of the population had used TCIM at least once (23). For almost half of the respondents, this experience was not longer than three years ago (47%; so-called current users); just under a fifth (19%) had undergone TCIM treatment several times during this period or had treated themselves. In this context, it is important to mention that in Switzerland Complementary Medicine is the dominant term for unconventional therapies. It should also be pointed out that within the given context, comparisons between societies of Germany and Switzerland can only be made to a limited extent due to different health policies and medical law regulations. In the present study, 70% stated that they had used TCIM at some point in their lives and 32% had made use of TCIM in the last 12 months. However, in Härtel/Volger 2004, exercise therapy (e.g., endurance training, targeted muscle training and physiotherapy were listed in the questionnaire) and massage were also asked about as TCIM interventions. This may explain the higher 12-month prevalence rates (11). This shows another general terminological problem in the field of TCIM—namely which interventions (in this country) are to be defined as TCIM interventions at all and which, for example, are already considered part of conventional medicine in Germany or are socially perceived as such, which can be very challenging, especially for participants in surveys. As diverse as the key terms in the spectrum of unconventional forms of diagnosis and therapy are in the German context (see introduction), a system for clearly defining and differentiating these methods is just as inconsistent in this country.

At the same time, certain therapeutic procedures that were considered questionable or dubious not so long ago are now often part of everyday practice in established medical settings (e.g., mindfulness techniques). This processual integration of individual TCIM procedures depends on the respective evidence base, including the increasing inclusion in medical guidelines; but probably also depends on reporting in leading German-language media (24).

There is also limited comparability with previous studies regarding indication based TCIM applications. The most common health problems for which TCIM was used in Härtel/Volger 2004 were back pain (57% of users), colds (29%), headaches (19%), fatigue (15%) and gastrointestinal complaints (12%)—where the question was asked for which complaints/diseases the most recently used TCIM treatment was (11). In this study, the participants had mainly used TCIM for musculoskeletal pain, followed by allergies, headaches, and mental illnesses. Musculoskeletal disorders were also mentioned in the first place in other previous studies (25, 26).

A high popularity of so-called, but not clearly defined herbal medicines (i.e., phytotherapeutics) can be assumed based on data from six German representative Allensbach surveys conducted between 1970 and 2010, with unified and standardized items (10). In 2010, almost three quarters of all Germans over the age of 16 had experience with herbal medicines (70%), a significantly higher proportion compared to 1970, when this only applied to half of the respondents (52%). Also, most recently (2010), 70% of respondents (1,882 people aged 16 and over) stated that they had used natural remedies at least once (10). In the present study, 42% took dietary supplements, vitamins or herbal remedies daily at the time of the survey. Due to the different wording of the questions, this aspect is only comparable to a very limited extent and represents a limitation of this survey.

In summary, it should be noted that most of the above-mentioned previous studies have not been published in peer-reviewed journals and were not conducted in cooperation with scientific university working groups. This means that the surveys carried out to date on this topic have only limited informative value. The present study is the first to provide up-to-date and reliable data on the use and acceptance of TCIM in Germany.

In Germany, the terms Complementary Medicine and Integrative Medicine are now established in the scientific medical community, but, above all, also in academic and tertial education contexts. However, this data set suggests that in contrast to this, the terms TEM and Alternative Medicine are used much more frequently in the German population for the same subject area. While this phenomenon should be subject of further transdisciplinary research, we see the most important need for action in closing the gap existing in social discourse regarding the generic terms used to ensure a standardized terminological basis for all those involved in the subject area (27). In view of the results, it should be questioned whether it would not be more meaningful to prefer the German term Naturheilkunde or the WHO term Traditional Medicine in academic medical work as well, instead of academically developed constructs that are considerably less well known among patients and users and are hardly used in society as a whole in Germany. Other representative surveys from other countries use a variety of other abbreviations for TCIM, e.g., traditional, complementary, and alternative medicine (TC&AM) or complementary and integrative health (CIH) (28–30). Standardization would be desirable here, e.g., as part of a Delphi method (31).

### 4.3 Future research

TCIM is attracting great interest internationally. A systematic review conducted as part of the EU FP7 CAMbrella project showed the use of TCIM varying between 0.3 and 86% in different EU countries, although the quality of the 87 studies included in this review varied considerably (25). However, the importance of TCIM in Europe is shown by the fact that, according to another CAMbrella study, there are around 305,000 registered doctors and therapists in Europe who offer these procedures, with more than half of these providers (approximately 160,000) being alternative practitioners (32). Due to the poor evidence base for most TCIM therapies, the CAMbrella Research Roadmap recommends a research strategy with sufficient funding.

The majority of Europeans want TCIM interventions as part of healthcare, but access is difficult in many countries, e.g., due to a lack of cost coverage by health insurance providers, a lack of services or insufficiently regulated qualifications of providers (33).

The WHO has been interested in TCIM for a long time. The WHO Traditional Medicine Program was launched in the 1970s. Since then, two global strategies on traditional medicine have been developed and a new one is planned for 2025–34 (2). There are several WHO guidelines for herbal remedies as well as for training in Ayurveda and traditional Chinese medicine; similar ones are planned for anthroposophic medicine, cupping and Tibetan medicine (34–36). At least 170 countries worldwide have documented the use of TCIM, and about 100 countries have national policies and programs, implying integration into the health care system, including Germany (3). In the official WHO strategy paper “Traditional Medicine 2014–2023” and at the 1st WHO Global Summit on Traditional Medicine in August 2023, the WHO also calls for the increased and consistent use of these methods in primary care (2–4).

In the last few decades particularly, the US National Institutes of Health (NIH) has systematically promoted research within the framework of the National Center for Complementary and Integrative Health (NCCIH) with the aim of generating further evidence for complementary and integrative medical interventions and supplementing existing strategies as part of comprehensive health management (3, 37). Although several larger research projects have been initiated in Germany in the last three decades (38–41), more intensive research often fails due to problematic funding.

TCIM research in Germany is hardly funded by the public sector, which contrasts with the frequent use by the population and the high popularity of TCIM and should therefore be highly relevant for medicine as a whole (33, 42). Currently, many TCIM interventions are not or are not sufficiently scientifically evaluated—this can also pose a potential risk to the population that should not be underestimated.

According to the data from this study, most participants are in favor of comprehensive funding for such projects in Germany as well. In the future, further evaluations of different integrative medical procedures and TCIM interventions should be carried out in the various healthcare sectors (26, 43).

## 5 Conclusion

Overall, the results of this online survey in a large representative sample indicates a high use and acceptance of TCIM in Germany. Therefore, the importance and relevance of TCIM in the German health care system must be given greater consideration regarding healthcare policy making. Considering the fact that the scientific evidence base of TCIM interventions must be strengthened, these procedures should be scientifically investigated in a systematic and rigorous manner utilizing high quality methodology, investigating their efficacy, effectiveness, therapeutic safety and costs of TCIM interventions.

## Data availability statement

The raw data supporting the conclusions of this article will be made available by the authors, without undue reservation.

## Ethics statement

The studies involving humans were approved by Charité University ethics committee. The studies were conducted in accordance with the local legislation and institutional requirements. The participants provided their written informed consent to participate in this study.

## Author contributions

MJ: Funding acquisition, Investigation, Project administration, Visualization, Writing – original draft. MO: Methodology, Writing – review & editing. BB: Supervision, Writing – review & editing. MS: Writing – review & editing. RH: Methodology, Project–administration, Supervision, Writing – review & editing. MT: Methodology, Writing – review & editing. AM: Supervision, Writing – review & editing. MW: Data curation, Formal analysis, Methodology, Software, Visualization, Writing – review & editing. CK: Conceptualization, Funding acquisition, Investigation, Project administration, Supervision, Writing – review & editing.

## In memoriam

The authors dedicate this article to Boike Rehbein (†11.6.2022). Boike Rehbein was involved in the planning and initiation of the project and sadly passed away during the project.
