# Supplementary material for: Use and acceptance of traditional, complementary and integrative medicine in Germany—an online representative cross-sectional study
Source: Front Med (Lausanne). 2024 Mar 13;11:1372924. doi: 10.3389/fmed.2024.1372924 (PMC10965565; doi:10.3389/fmed.2024.1372924)
Supplement: Supplementary file 2 [file Data_Sheet_2.docx]

Supplementary Material 2

**Use and Acceptance of Traditional, Complementary and Integrative Medicine (TCIM) in Germany – an Online Representative Cross-sectional Study**

**Supplementary Table 1:** Sociodemographic characteristics of TCIM use in the last 12 months

| **TCIM use in the last 12 months** | **unweighted** | | | | | | **weighted** | | | | | | | | | | | | |
| --- | --- | --- | --- | --- | --- | --- | --- | --- | --- | --- | --- | --- | --- | --- | --- | --- | --- | --- | --- |
|  | **All (n=4065)** | | **Yes (n= 1291; 31.8%)** | **No (n= 2572; 63.4%)** | **I don’t know (n=202; 5%)** | **p-value** | | **All (n=4065)** | | | | **Yes (n= 1293; 31.8%)** | **No (n= 2554; 62.8%)** | **I don´t know (n=219; 5.4%)** | | | **p-value** | | |
|  | n | % | % | % | % |  | | n | % | | % | | % | | % | | |  | |
| ***Gender*** |  |  |  |  |  |  | |  |  | |  | |  | |  | | |  | |
| Male | 1947 | 47.9 | 39.1 | 52.4 | 46 | < 0.001 | | 2026 | 49.8 | | 41.5 | | 54.2 | | 47.5 | | | < 0.001 | |
| Female | 2101 | 51.7 | 60.7 | 47.1 | 52.5 |  |  | 2018 | 49.6 | | 58.2 | | 45.2 | | 507 | | |  |  |
| Diverse | 17 | 0.4 | 0.2 | 0.4 | 1.5 |  |  | 21 | 0.5 | | 0.3 | | 0.5 | | 1.8 | | |  |  |
| ***Age in years*** |  |  |  |  |  |  | |  | | | | | | | | | | | |
| Under 20 | 67 | 1.6 | 2 | 1.2 | 4.5 | < 0.001 | | 87 | 2.1 | | 2.4 | | 1.8 | | 5.0 | | | < 0.001 | |
| 20 to 29 | 557 | 13.7 | 13.9 | 12.8 | 24.3 |  |  | 679 | 16.7 | | 16.7 | | 15.7 | | 28.0 | | |  |  |
| 30 to 39 | 631 | 15.5 | 17 | 14.8 | 15.8 |  |  | 701 | 17.2 | | 18.6 | | 16.6 | | 17.0 | | |  |  |
| 40 to 49 | 656 | 16.1 | 18.3 | 14.9 | 18.3 |  |  | 651 | 16.0 | | 18.0 | | 14.8 | | 18.3 | | |  |  |
| 50 to 59 | 896 | 22 | 20.4 | 23 | 19.8 |  |  | 863 | 21.2 | | 19.6 | | 22.2 | | 18.8 | | |  |  |
| 60 to 75 | 1258 | 30.9 | 28.4 | 33.3 | 17.3 |  |  | 1084 | 26.7 | | 24.7 | | 28.9 | | 12.8 | | |  |  |
| ***Education*** |  |  |  |  |  |  | |  |  | |  | |  | |  | | |  | |
| No general school-leaving certificate (yet), still a pupil at a general school | 30 | 0.7 | 0.4 | 0.7 | 3.5 | < 0.001 | | 36 | 0.9 | | 0.5 | | 0.8 | | 4.1 | | | < 0.001 | |
| Secondary (elementary, basic) school leaving certificate without completed apprenticeship/vocational training | 251 | 6.2 | 3.7 | 7.4 | 6.4 |  |  | 271 | 6.7 | | 4.1 | | 7.9 | | 7.3 | | |  |  |
| Secondary school leaving certificate with completed apprenticeship/vocational training | 885 | 21.8 | 17.3 | 24.1 | 20.8 |  |  | 891 | 21.9 | | 17.8 | | 24.2 | | 19.7 | | |  |  |
| Secondary school without A-levels (German: Realschulabschluss/Mittlere Reife/Oberschule) or equivalent qualification | 1173 | 28.9 | 29.6 | 28.7 | 26.2 |  |  | 1288 | 31.7 | | 32.9 | | 31.3 | | 28.9 | | |  |  |
| A-levels, (technical) university entrance qualification without studies | 751 | 18.5 | 20.3 | 17.3 | 21.8 |  |  | 723 | 17.8 | | 19.9 | | 16.5 | | 21.1 | | |  |  |
| Studies (university, college, university of applied sciences, polytechnic) | 949 | 23.3 | 27.7 | 21.3 | 21.3 |  |  | 835 | 20.6 | | 24.1 | | 18.9 | | 18.8 | | |  |  |
| PhD | 26 | 0.6 | 1.0 | 0.5 | 0.0 |  |  | 19 | 0.5 | | 0.7 | | 0.4 | | 0.0 | | |  |  |
| ***Federal states*** | | | | | | |  | |  |  | | |  | | |  | | |  |
| Baden-Württemberg | 417 | 10.3 | 11.9 | 9.6 | 8.4 | 0.263 | | 537 | 13.2 | | 14.9 | | 12.4 | | 12.3 | | | 0.057 | |
| Bavaria | 604 | 14.9 | 15.3 | 14.5 | 16.3 |  |  | 630 | 15.5 | | 16.3 | | 15.0 | | 16.0 | | |  |  |
| Berlin | 342 | 8.4 | 7.8 | 8.6 | 9.9 |  |  | 174 | 4.3 | | 4.1 | | 4.3 | | 4.8 | | |  |  |
| Brandenburg | 98 | 2.4 | 1.6 | 2.8 | 2.0 |  |  | 122 | 3 | | 2.1 | | 3.5 | | 2.3 | | |  |  |
| Bremen | 40 | 1 | 0.9 | 1.0 | 0.5 |  |  | 33 | 0.8 | | 0.8 | | 0.9 | | 0.5 | | |  |  |
| Hamburg | 159 | 3.9 | 3.3 | 4.3 | 3.5 |  |  | 89 | 2.2 | | 1.8 | | 2.4 | | 1.8 | | |  |  |
| Hesse | 293 | 7.2 | 7.8 | 7.0 | 5.4 |  |  | 305 | 7.5 | | 8.4 | | 7.2 | | 5.9 | | |  |  |
| Mecklenburg-Western Pomerania | 71 | 1.7 | 1.8 | 1.6 | 3.0 |  |  | 78 | 1.9 | | 1.9 | | 1.8 | | 3.7 | | |  |  |
| Lower Saxony | 341 | 8.4 | 6.7 | 9.2 | 8.4 |  |  | 403 | 9.9 | | 7.6 | | 11.2 | | 9.1 | | |  |  |
| North Rhine-Westphalia | 868 | 21.4 | 22.5 | 20.8 | 21.8 |  |  | 878 | 21.6 | | 22.5 | | 21.0 | | 22.4 | | |  |  |
| Rhineland-Palatinate | 179 | 4.4 | 3.6 | 4.6 | 6.9 |  |  | 203 | 5 | | 4.1 | | 5.1 | | 8.7 | | |  |  |
| Saarland | 43 | 1.1 | 1.0 | 1.1 | 1.0 |  |  | 49 | 1.2 | | 1.1 | | 1.3 | | 0.9 | | |  |  |
| Saxony | 238 | 5.9 | 6.1 | 5.9 | 4.0 |  |  | 199 | 4.9 | | 5.1 | | 4.9 | | 3.2 | | |  |  |
| Saxony-Anhalt | 107 | 2.6 | 2.9 | 2.5 | 2.0 |  |  | 106 | 2.6 | | 2.9 | | 2.5 | | 1.8 | | |  |  |
| Schleswig-Holstein | 152 | 3.7 | 3.7 | 3.7 | 4.0 |  |  | 146 | 3.6 | | 3.5 | | 3.6 | | 4.1 | | |  |  |
| Thuringia | 113 | 2.8 | 3.0 | 2.6 | 3.0 |  |  | 114 | 2.8 | | 3.0 | | 2.7 | | 2.7 | | |  |  |
| ***Personal monthly net income*** | | | | | | |  | |  |  | | |  | | |  | | |  |
| No own income | 175 | 4.3 | 3.6 | 4.4 | 6.9 | 0.37 | | 199 | 4.9 | | 4.0 | | 5.0 | | 8.3 | | | 0.037 | |
| Up to 1000 € | 887 | 21.8 | 21.3 | 21.6 | 28.2 |  |  | 883 | 21.7 | | 21.4 | | 21.4 | | 27.5 | | |  |  |
| 1000-2000 € | 1549 | 38.1 | 38 | 38.2 | 38.1 |  |  | 1544 | 38 | | 38.7 | | 37.9 | | 35.3 | | |  |  |
| 2000-3000 € | 959 | 23.6 | 23.7 | 24 | 18.3 |  |  | 952 | 23.4 | | 23.2 | | 23.9 | | 19.3 | | |  |  |
| 3000-4000 € | 311 | 7.7 | 8.1 | 7.7 | 4.5 |  |  | 317 | 7.8 | | 7.8 | | 7.9 | | 6.0 | | |  |  |
| 4000-5000 € | 99 | 2.4 | 2.9 | 2.3 | 0.5 |  |  | 93 | 2.3 | | 2.7 | | 2.2 | | 0.5 | | |  |  |
| > 5000 € | 85 | 2.1 | 2.4 | 1.8 | 3.5 |  |  | 76 | 1.9 | | 2.2 | | 1.6 | | 3.2 | | |  |  |
| ***Net monthly household income*** | | | | | | |  | |  |  | | |  | | |  | | |  |
| Up to 1000 € | 505 | 12.4 | 10.3 | 13.2 | 17.3 | < 0.001 | | 482 | 11.9 | | 9.4 | | 12.6 | | 18.3 | | | < 0.001 | |
| 1000-2000 € | 1047 | 25.8 | 22.5 | 26.7 | 33.7 |  |  | 1023 | 25.2 | | 22.6 | | 26.1 | | 28.9 | | |  |  |
| 2000-3000 € | 1049 | 25.8 | 26.5 | 25.7 | 22.8 |  |  | 1051 | 25.8 | | 27.1 | | 25.5 | | 22.9 | | |  |  |
| 3000-4000 € | 735 | 18.1 | 20.1 | 17.2 | 16.3 |  |  | 769 | 18.9 | | 20.5 | | 18.0 | | 20.2 | | |  |  |
| 4000-5000 € | 424 | 10.4 | 12.5 | 9.8 | 4.5 |  |  | 443 | 10.9 | | 12.5 | | 10.7 | | 4.1 | | |  |  |
| > 5000 € | 305 | 7.5 | 8.1 | 7.3 | 5.4 |  |  | 298 | 7.3 | | 8.0 | | 7.2 | | 5.5 | | |  |  |
| ***Location size*** | | | | | | |  | |  |  | | |  | | |  | | |  |
| Under 2,000 inhabitants | 273 | 6.7 | 6.9 | 6.5 | 8.4 | 0.829 | | 310 | 7.6 | | 7.7 | | 7.4 | | 10.1 | | | 0.745 | |
| 2,000 to under 5,000 inhabitants | 232 | 5.7 | 6 | 5.4 | 7.9 |  |  | 267 | 6.6 | | 6.7 | | 6.3 | | 8.7 | | |  |  |
| 5,000 to under 20,000 inhabitants | 603 | 14.8 | 14.4 | 14.9 | 16.3 |  |  | 1072 | 26.4 | | 25.8 | | 26.5 | | 28.4 | | |  |  |
| 20,000 to under 50,000 inhabitants | 557 | 13.7 | 13.6 | 13.6 | 14.9 |  |  | 655 | 16.1 | | 15.8 | | 16.3 | | 16.1 | | |  |  |
| 50,000 to under 100,000 inhabitants | 401 | 9.9 | 10.1 | 9.8 | 9.9 |  |  | 470 | 11.6 | | 12.1 | | 11.4 | | 10.6 | | |  |  |
| 100,000 to under 500,000 inhabitants | 889 | 21.9 | 22.2 | 22.2 | 16.3 |  |  | 617 | 15.2 | | 15.4 | | 15.5 | | 10.6 | | |  |  |
| 500,000 inhabitants and more | 1110 | 27.3 | 26.9 | 27.6 | 26.2 |  |  | 674 | 16.6 | | 16.5 | | 16.7 | | 15.6 | | |  |  |
| ***Religious community*** | | | | | | |  | |  |  | | |  | | |  | | |  |
| Catholic | 853 | 21.0 | 22.9 | 19.9 | 23.3 | < 0.001 | | 894 | 22.0 | | 23.9 | | 20.8 | | 24.2 | | | < 0.001 | |
| Protestant | 1051 | 25.9 | 25.3 | 26.4 | 23.3 |  |  | 1096 | 27.0 | | 25.9 | | 27.7 | | 24.7 | | |  |  |
| Muslim | 69 | 1.7 | 1.9 | 1.5 | 3.5 |  |  | 70 | 1.7 | | 1.8 | | 1.5 | | 4.1 | | |  |  |
| Buddhist | 17 | 0.4 | 0.8 | 0.2 | 0.5 |  |  | 18 | 0.4 | | 0.9 | | 0.2 | | 0.5 | | |  |  |
| Hindu | 2 | 0.1 | 0.2 | 0 | 0 |  |  | 2 | 0.1 | | 0.2 | | 0.0 | | 0.0 | | |  |  |
| Jewish | 12 | 0.3 | 0.7 | 0.1 | 0.5 |  |  | 8 | 0.2 | | 0.5 | | 0.0 | | 0.5 | | |  |  |
| Other | 72 | 1.8 | 2.6 | 1.4 | 2 |  |  | 68 | 1.7 | | 2.4 | | 1.3 | | 2.3 | | |  |  |
| No religious affiliation/atheist | 1989 | 48.9 | 45.9 | 50.6 | 47 |  |  | 1909 | 46.9 | | 44.5 | | 48.5 | | 43.8 | | |  |  |
| ***Party affiliation*** | | | | | | |  | |  |  | | |  | | |  | | |  |
| CDU | 506 | 12.4 | 13.8 | 12 | 9.4 | < 0.001 | | 515 | 12.7 | | 13.6 | | 12.5 | | 9.6 | | | 0.001 | |
| CSU | 177 | 4.4 | 4.7 | 4.3 | 2.5 |  |  | 184 | 4.5 | | 5.0 | | 4.4 | | 3.2 | | |  |  |
| FDP | 229 | 5.6 | 5.7 | 5.6 | 5 |  |  | 244 | 6 | | 5.9 | | 6.1 | | 5.0 | | |  |  |
| Die Grüne | 637 | 15.7 | 16.6 | 15.2 | 15.8 |  |  | 595 | 14.6 | | 15.0 | | 14.5 | | 13.7 | | |  |  |
| SPD | 650 | 16 | 16.2 | 16.4 | 9.9 |  |  | 629 | 15.5 | | 16.3 | | 15.5 | | 10.0 | | |  |  |
| Linke | 330 | 8.1 | 7.6 | 8.6 | 5 |  |  | 311 | 7.6 | | 7.3 | | 8.1 | | 4.6 | | |  |  |
| AFD | 418 | 10.3 | 10.1 | 10.2 | 11.9 |  |  | 443 | 10.9 | | 11.0 | | 10.7 | | 12.3 | | |  |  |
| Other political parties | 194 | 4.8 | 5.7 | 4.4 | 4.5 |  |  | 206 | 5.1 | | 5.8 | | 4.7 | | 5.0 | | |  |  |
| Not specified | 924 | 22.7 | 19.6 | 23.3 | 36.1 |  |  | 941 | 23.1 | | 20.0 | | 23.6 | | 36.5 | | |  |  |
| ***Medical background*** | | | | | | |  | |  |  | | |  | | |  | | |  |
| Nursing training/nursing studies | 195 | 4.8 | 6.4 | 4.1 | 4 | 0.007 | | 193 | 4.7 | | 6.2 | | 4.1 | | 4.1 | | | 0.013 | |
| Alternative practitioner examination | 39 | 1 | 2.5 | 0.3 | 0 | 0.001 | | 42 | 1 | | 2.4 | | 0.4 | | 0.0 | | | < 0.001 | |
| Medical studies | 51 | 1.3 | 1.6 | 1.1 | 1 | 0.345 | | 57 | 1.4 | | 1.5 | | 1.4 | | 1.4 | | | 0.969 | |
| Pharmacy studies | 29 | 0.7 | 1.2 | 0.5 | 0 | 0.018 | | 32 | 0.8 | | 1.2 | | 0.7 | | 0.0 | | | 0.104 | |
| Physiotherapy training | 39 | 1 | 1.7 | 0.6 | 0.5 | 0.004 | | 43 | 1.1 | | 1.6 | | 0.9 | | 0.0 | | | 0.027 | |
| Occupational therapy training | 27 | 0.7 | 1.2 | 0.4 | 0.5 | 0.029 | | 27 | 0.7 | | 0.9 | | 0.6 | | 0.5 | | | 0.591 | |
| Self-acquisition of basic medical knowledge | 670 | 16.5 | 25.9 | 11.9 | 15.3 | < 0.001 | | 670 | 16.5 | | 26.2 | | 11.7 | | 14.7 | | | < 0.001 | |
| Miscellaneous | 112 | 2.8 | 3.8 | 2.2 | 3.5 | 0.012 | | 110 | 2.7 | | 3.6 | | 2.2 | | 4.1 | | | 0.017 | |

**
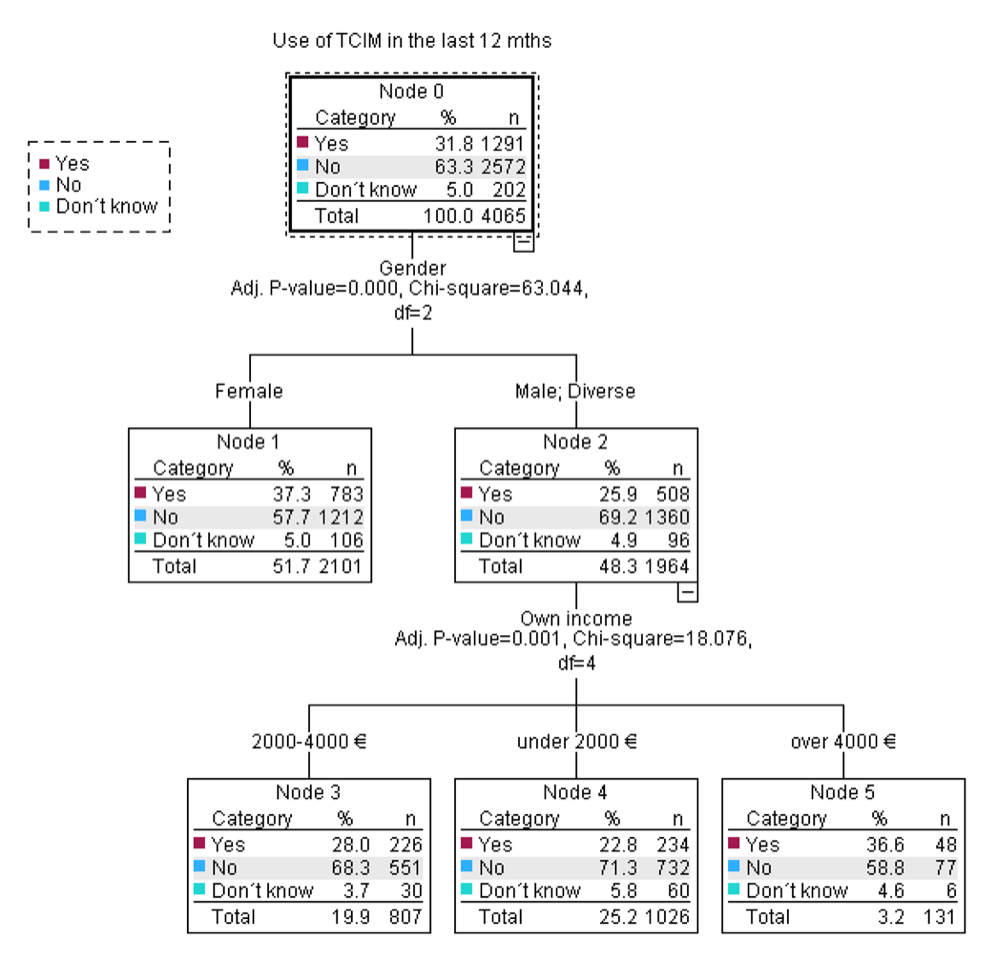
**

**Supplementary Figure 1:** Decision tree for TCIM use in the last 12 months for gender and own monthly net income

**Supplementary Figure 2:** To what extent are you familiar with these medical procedures?

**Supplementary Figure 3:** How much do the aspects listed below influence your decision to choose a treatment method (Likert scale 1-5 from 1= does not influence my decision at all to 5= influences my decision enormously or extremely strongly)?
